# Involvement of the pyroptosis–HMGB1 axis in systemic diseases

**DOI:** 10.3389/fcell.2026.1814812

**Published:** 2026-06-01

**Authors:** Ting Wang, Le Zhang, Jian Du, Qiuli Miao

**Affiliations:** 1 Department of General Surgery, Lequn Branch, The First Hospital of Jilin University, Changchun, China; 2 Department of Dermatology, The Affiliated Hospital of Changchun University of Traditional Chinese Medicine, Changchun, China; 3 Department of Endocrinology and Spleen-Stomach Diseases, The Third Affiliated Hospital of Changchun University of Chinese Medicine, Changchun, China; 4 Department of Pharmacy, Lequn Branch, The First Hospital of Jilin University, Changchun, China

**Keywords:** gasdermins, HMGB1, inflammasomes, pyroptosis, regulated cell death, systemic diseases

## Abstract

Pyroptosis is a lytic form of regulated cell death driven by inflammasome activation and gasdermin-mediated membrane rupture, characterized by robust inflammatory amplification. High-mobility group box 1 (HMGB1), a multifunctional nuclear protein and damage-associated molecular pattern, has emerged as a central regulator within pyroptosis-associated pathologies. Acting both as a downstream alarmin released during pyroptotic cell death and as an upstream licensing factor for inflammasome activation and innate immune signaling, HMGB1 establishes a context-dependent feedforward loop that shapes tissue injury and immune responses. In infectious and inflammatory disorders, excessive HMGB1 release exacerbates immune dysregulation and organ damage, whereas in selected tumor settings, pyroptosis-associated HMGB1 contributes to immunogenic cell death and antitumor immunity. This review systematically summarizes the molecular mechanisms underlying HMGB1-regulated pyroptosis, including canonical and noncanonical inflammasome pathways, gasdermin execution, and crosstalk with other regulated cell death programs. We further integrate evidence implicating the pyroptosis–HMGB1 axis across systemic diseases involving the nervous, respiratory, digestive, circulatory, urinary, locomotor, endocrine, reproductive, and immune systems. Finally, we discuss emerging therapeutic strategies targeting this axis, highlighting opportunities and challenges for disease-specific and precision interventions.

## 1 Introduction

Pyroptosis is a form of regulated cell death (RCD) distinguished by gasdermin-mediated membrane permeabilization and its intrinsically pro-inflammatory outcome (Lage et al., 2013). Unlike apoptosis, which preserves membrane integrity and is largely immunologically silent, pyroptosis culminates in rapid cell swelling, membrane rupture, and the release of inflammatory mediators and damage-associated molecular patterns (DAMPs), including interleukin (IL)-1β, IL-18, ATP, and high mobility group box 1 (HMGB1) (Zhang and Wirtz, 2022; Wen et al., 2023; Stephenson et al., 2016). Although morphologically lytic, pyroptosis is not a passive necrotic process but an actively orchestrated program governed by intracellular danger sensing, inflammasome assembly, and caspase-dependent gasdermin cleavage (Zhang and Wirtz, 2022).

At the molecular level, canonical pyroptosis is initiated by cytosolic pattern recognition receptors (PRRs), such as NLR family pyrin domain-containing 3 (NLRP3), NLR family CARD domain-containing 4 (NLRC4), and absent in melanoma 2 (AIM2), which assemble inflammasome complexes to activate inflammatory caspases, primarily caspase-1, leading to cleavage of gasdermin D (GSDMD) and pore formation in the plasma membrane (Lage et al., 2013; Linder et al., 2020; Yang et al., 2022). Non-canonical pathways mediated by caspase-4/5 in humans (caspase-11 in mice) and alternative execution routes involving caspase-3–gasdermin E (GSDME) or granzyme-dependent mechanisms further expand pyroptosis beyond inflammasome-restricted models (Wen et al., 2023; Bai et al., 2025). Importantly, pyroptotic susceptibility and execution are highly cell type–dependent; for example, neutrophils display distinct regulatory features and relative resistance to canonical inflammasome-driven lysis, underscoring functional heterogeneity within the innate immune system (Sollberger, 2022).

Among the DAMPs liberated during pyroptosis, HMGB1 has emerged as a central mediator that couples inflammatory cell death to immune amplification. HMGB1 is a ubiquitously expressed nuclear protein that regulates chromatin organization under homeostatic conditions but acquires potent immunostimulatory functions upon cytoplasmic translocation and extracellular release. Extracellular HMGB1 signals through receptors such as advanced glycosylation end product-specific receptor (RAGE) and Toll-like receptors (TLR2 and TLR4), thereby amplifying innate immune responses (Chen R. C. et al., 2022; Yang et al., 2015). Beyond acting as a soluble ligand, HMGB1 functions as an “alarmin shuttle”: by forming complexes with co-released inflammatory cargos, including lipopolysaccharide (LPS), nucleic acids, and IL-1 family cytokines, HMGB1 facilitates their endocytic uptake and cytosolic delivery, enabling engagement of intracellular danger sensors that would otherwise remain inaccessible (Andersson et al., 2018).

Pyroptosis and HMGB1 are therefore linked through a bidirectional, self-reinforcing circuit. Inflammasome-driven pyroptosis is sufficient to induce rapid HMGB1 release independent of *de novo* transcription, positioning pyroptosis as a dominant mechanism of DAMP liberation (Nyström et al., 2013). Conversely, extracellular HMGB1 amplifies inflammatory signaling through RAGE- and TLR4-dependent pathways, promotes inflammasome activation, and facilitates non-canonical pyroptosis by enabling cytosolic delivery of extracellular pathogen-associated molecular patterns (PAMPs) such as LPS (Andersson et al., 2018; Lei et al., 2024). Notably, the inflammatory identity of released HMGB1 is shaped by priming signals, with surface TLR engagement favoring redox states that confer strong TLR4 agonistic activity (Nyström et al., 2013).

Collectively, these observations support the concept of a self-amplifying pyroptosis–HMGB1 axis, in which gasdermin-mediated lytic death drives HMGB1 release, while HMGB1 in turn sustains inflammatory signaling and inflammasome activation (Chen R. C. et al., 2022; Yang et al., 2015; Nyström et al., 2013; Lei et al., 2024; Liu and Lieberman, 2024). Dysregulation of this axis has been implicated across diverse pathological contexts, including systemic inflammatory disorders, metabolic disease, cancer, and neurodegeneration (Gao H. C. et al., 2025; Yang H. H. et al., 2024; Homma et al., 2024; Eldridge et al., 2017). In this review, we synthesize current mechanistic insights into pyroptotic signaling, examine the pathological roles of HMGB1-associated pyroptosis across organ systems, and discuss emerging therapeutic strategies targeting this inflammatory death axis.

## 2 Pyroptotic signaling pathways

**FIGURE 1:**
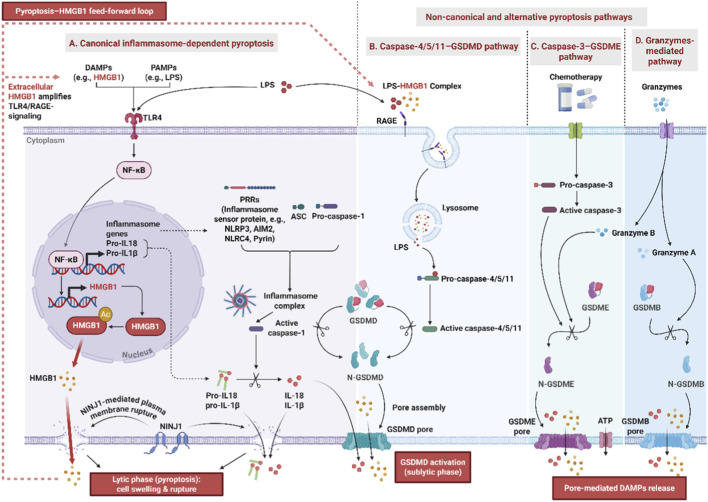
Overview of pyroptotic signaling pathways and HMGB1 release. Pyroptosis is executed through canonical inflammasome-dependent pathways and multiple inflammasome-independent or alternative routes that converge on gasdermin activation and plasma membrane permeabilization. Canonical pyroptosis is initiated by pattern recognition receptor–driven inflammasome assembly, leading to caspase-1 activation, gasdermin D (GSDMD) cleavage, cytokine maturation, and NINJ1-dependent membrane rupture with HMGB1 release. Non-canonical pyroptosis is triggered by cytosolic lipopolysaccharide (LPS) sensing via caspase-4/5/11, facilitated by HMGB1–LPS complexes and receptor for advanced glycation end products (RAGE)–mediated endocytosis. Alternative execution pathways include caspase-3–gasdermin E (GSDME) activation during chemotherapy and granzyme-mediated cleavage of gasdermins by cytotoxic lymphocytes. Depending on pore density and cellular context, gasdermin activation results in sublytic or lytic membrane damage, thereby shaping the magnitude of cytokine and damage-associated molecular pattern (DAMP) release, including HMGB1.

Pyroptosis can be initiated through multiple molecular routes that converge on gasdermin activation and membrane disruption. These pathways differ in upstream sensing mechanisms, protease usage, and qualitative patterns of cytokine maturation and DAMP release—features that critically shape HMGB1 liberation and inflammatory amplification. An integrated overview of these signaling routes is summarized in Figure 1.

### 2.1 Canonical inflammasome-dependent pyroptosis

Canonical pyroptosis is initiated by cytosolic PRRs, including NLR family members (e.g., NLRP3 and NLRC4), the pyrin inflammasome, and AIM2-like receptors, which detect diverse PAMPs/DAMPs and trigger inflammasome assembly (Zhang and Wirtz, 2022). These multiprotein platforms typically comprise a sensor PRR, the adaptor ASC, and pro–caspase-1. Sensor architecture dictates ASC dependence: PYD-containing receptors require ASC to recruit caspase-1, whereas CARD-containing sensors such as NLRC4 can directly engage caspase-1, with ASC functioning as an optional signal amplifier (Liu and Lieberman, 2024; Tsuchiya, 2020). This modularity establishes ASC oligomerization as a context-dependent regulatory checkpoint rather than an absolute requirement for pyroptotic execution (Darweesh et al., 2019; Van Opdenbosch et al., 2014).

Activated caspase-1 cleaves GSDMD, releasing its N-terminal pore-forming fragment that inserts into the plasma membrane and induces ionic imbalance, osmotic swelling, and inflammatory cell death (Linder et al., 2020; Liu and Lieberman, 2024). However, gasdermin pore formation alone is often insufficient for catastrophic membrane rupture. Pyroptotic cells typically require additional mechanical destabilization mediated by calpain-dependent cytoskeletal cleavage, particularly of vimentin, to progress to full lysis and release of large DAMPs such as HMGB1 (Volchuk et al., 2020). In this context, the cell-surface protein ninjurin-1 (NINJ1) has been identified as a key executor of terminal membrane rupture. While NINJ1-deficient cells undergo intact inflammasome activation, GSDMD cleavage, and IL-1β release, they fail to rupture and release HMGB1, positioning NINJ1 as a gatekeeper of lytic DAMP dissemination (Kayagaki et al., 2021; Davis et al., 2019).

Canonical inflammasome activation is tightly coupled to innate immune priming. TLR-mediated signaling induces nuclear factor kappa B (NF-κB)–dependent transcription of inflammasome components and pro–IL-1β, thereby licensing robust cytokine maturation upon caspase-1 activation (Lei et al., 2024). Importantly, pyroptosis itself is sufficient to drive rapid HMGB1 release, even in the absence of priming, underscoring lytic death as the dominant determinant of HMGB1 liberation (Volchuk et al., 2020). Priming instead modulates the redox state of released HMGB1, thereby shaping its downstream immunological activity (Nyström et al., 2013).

### 2.2 Inflammasome-independent and alternative pyroptosis pathways

Beyond canonical inflammasomes, several inflammasome-independent routes can execute pyroptosis by directly activating gasdermin family members, expanding pyroptosis into a broader category of gasdermin-mediated lytic death.

#### 2.2.1 Caspase-4/5/11–GSDMD pathway

In non-canonical pyroptosis, caspase-4/5 in humans and caspase-11 in mice directly sense cytosolic LPS via their CARD domains, leading to oligomerization and activation independent of upstream PRRs or adaptor proteins (Liu and Lieberman, 2024). These caspases cleave GSDMD, inducing rapid membrane permeabilization. Although they do not directly process IL-1β or IL-18, GSDMD-mediated potassium efflux secondarily activates the NLRP3 inflammasome, amplifying cytokine maturation (Liu and Lieberman, 2024; Wright et al., 2022; Downs et al., 2020; Tian et al., 2024).

Non-canonical pyroptosis is particularly effective at driving HMGB1 release. Notably, systemic HMGB1 elevation during endotoxemia persists even when IL-1β secretion is impaired, highlighting lytic death rather than selective secretion as the primary determinant of HMGB1 release (Volchuk et al., 2020). HMGB1 further reinforces this pathway by acting as an extracellular shuttle for LPS, promoting its RAGE-dependent endocytosis and lysosomal escape, thereby enabling cytosolic activation of caspase-11/4/5 (Andersson et al., 2018; Kim and Kim, 2018).

#### 2.2.2 Caspase-3–GSDME–mediated apoptosis–pyroptosis switch

An important alternative route links apoptotic signaling to pyroptotic execution through caspase-3–mediated cleavage of GSDME. Robust caspase-3 activation converts a classically non-lytic apoptotic program into inflammatory membrane rupture with ATP and HMGB1 release (Zhang and Wirtz, 2022; Wen et al., 2023). This mechanism is particularly prominent in cancer therapy, where chemotherapy-induced caspase-3/GSDME activation enhances immunogenic cell death. Similar execution logic operates in innate immune cells, where ATP-induced lysis proceeds through caspase-3–GSDME rather than inflammasome-dependent caspase-1 activation (Zeng et al., 2019).

#### 2.2.3 Granzymes-mediated pyroptosis

Cytotoxic lymphocytes can directly trigger gasdermin activation through granzyme delivery. Granzyme B cleaves GSDME, whereas granzyme A activates GSDMB, bypassing inflammasome signaling entirely (Wen et al., 2023; Yang et al., 2022). This mode of pyroptosis couples immune cytotoxicity to inflammatory DAMP release, including HMGB1, and reshapes local immune microenvironments. Disease models, including viral hepatitis–associated liver failure, support a pathogenic role for granzyme-driven pyroptosis and HMGB1 release in inflammatory tissue injury (Ning et al., 2022; Zhao Q. et al., 2025).

Despite distinct upstream triggers and proteolytic machineries, canonical and alternative pyroptosis pathways converge on gasdermin-mediated membrane damage and lytic HMGB1 release. Differences in sensing, execution, and priming determine the qualitative inflammatory output, but all routes ultimately feed into a shared pyroptosis–HMGB1 amplification loop. This mechanistic convergence provides a unifying framework for understanding how localized regulated cell death escalates into sustained inflammatory responses that drive systemic disease pathology.

## 3 Systemic landscape of the pyroptosis–HMGB1 axis across human diseases

### 3.1 Nervous system diseases

#### 3.1.1 Acute central nervous system (CNS) injury

Acute CNS injury typically follows a two-phase trajectory: an initial insult triggers rapid tissue disruption, followed by HMGB1-centered secondary inflammatory amplification. Within this cascade, pyroptosis contributes to blood–brain barrier (BBB) breakdown, innate immune activation, and progressive tissue loss, thereby linking local cell death to system-level neuroinflammation.

In intracerebral hemorrhage (ICH), peri-hematomal inflammation is rapidly initiated by DAMP release and inflammasome engagement. Experimental inhibition of HMGB1 or TLR4 ameliorates neurological deficits and suppresses canonical pyroptosis signaling, including inflammasome assembly, caspase-1 activation, IL-1β maturation, and GSDMD cleavage. Notably, HMGB1/TLR4 blockade preferentially attenuates NLRP3 activation, supporting an HMGB1–TLR4–NLRP3 axis as a dominant conduit linking hemorrhagic injury to caspase-1–dependent pyroptosis and inflammatory amplification (Lei et al., 2024).

While hemorrhage highlights DAMP-triggered inflammasome activation, ischemia–reperfusion injury emphasizes upstream metabolic licensing of this axis. A metabolic–epigenetic circuit has been described whereby lactate dehydrogenase A (LDHA)-driven lactate accumulation increases histone lactylation at the HMGB1 promoter, elevates HMGB1 expression, and enables NLRP3/caspase-1/GSDMD activation in oxygen–glucose deprivation/reoxygenation models; HMGB1 overexpression restores pyroptosis even when LDHA is silenced, placing HMGB1 downstream of metabolic rewiring in ischemic injury (Yao and Li, 2023). In line with this hierarchy, interventions that restrain HMGB1 and/or inflammasome activation consistently reduce infarct burden, inflammatory cytokine production, and BBB injury across ischemic models (Zhang Y. H. et al., 2025; Liang et al., 2021).

In traumatic brain injury (TBI), the same HMGB1–pyroptosis circuitry can be embedded within a vascular–immune feed-forward loop. Neutrophil extracellular traps (NETs) induce endothelial pyroptosis via TLR4/NF-κB signaling, while NINJ1-dependent terminal membrane rupture facilitates HMGB1 release; extracellular HMGB1 in turn promotes NET formation, collectively exacerbating BBB disruption and neurological injury. Targeting NINJ1 interrupts this loop and improves functional outcomes (Zheng X. B. et al., 2025), underscoring that HMGB1 release may actively reinforce neurovascular inflammation rather than serving solely as a passive consequence of lytic death.

Beyond the brain, spinal cord injury (SCI) and spinal ischemia–reperfusion injury (SCI/R) further illustrate post-transcriptional control of the HMGB1–inflammasome node. In SCI/R, long non-coding RNA (lncRNA) H19 derepresses HMGB1 via microRNA (miR)-181a-5p sponging, thereby enhancing NLRP3/caspase-1 activation and pyroptosis; silencing H19 reduces inflammatory injury and improves locomotor recovery (Guo et al., 2022). Taken together, acute CNS and spinal injuries converge on HMGB1-enabled inflammasome execution, while differing in the upstream “licensing” inputs—DAMP release, metabolic rewiring, neurovascular feed-forward loops, and non-coding RNA regulation. Taken together, acute CNS and spinal injuries converge on HMGB1-enabled inflammasome execution, while differing in upstream licensing inputs (DAMP release, metabolic rewiring, neurovascular feed-forward loops, and non-coding RNA regulation). This axis is consistently supported across injury, neurodegeneration, and pain models, yet several caveats limit translational inference.

#### 3.1.2 Chronic neuroinflammatory and neurodegeneration-related disorders

In chronic neurological disorders, pyroptosis-related signaling is more closely linked to persistent neuroinflammation and progressive dysfunction than to acute tissue destruction, often sustained by recurrent HMGB1 release and inflammasome priming. In depression-related models, stressors activate hippocampal HMGB1/RAGE/NLRP3 signaling and GSDMD-dependent injury in neural lineages, while anti-inflammatory interventions suppress inflammasome activation, reduce HMGB1 release, and ameliorate behavioral abnormalities (Yang et al., 2020; Chen Y. P. et al., 2022). In Parkinson’s disease models, dampening pyroptosis-associated signaling similarly attenuates glial inflammation and dopaminergic neuron loss, accompanied by modulation of HMGB1-linked pathways, suggesting that HMGB1–inflammasome coupling may represent a shared inflammatory module across neurodegenerative contexts (Saad et al., 2025). More broadly, atypical necrotic programs in neurodegeneration may perpetuate HMGB1 externalization and chronic inflammation, reinforcing a paradigm in which sustained DAMP signaling drives maladaptive neuroimmune activation (Homma et al., 2024).

#### 3.1.3 Systemic inflammation/metabolic stress–associated neurological complications

Neurological dysfunction can also arise secondarily from systemic inflammatory or metabolic disease, highlighting peripheral–central immune crosstalk mediated by HMGB1 and inflammasome-dependent pyroptosis. In sepsis-associated encephalopathy, mesenchymal stem cell (MSC)-derived exosomal miR-140-3p directly targets HMGB1, suppresses NLRP3/caspase-1/GSDMD activation, and mitigates cognitive impairment, underscoring extracellular vesicle (EV)-mediated regulation of systemic-to-brain inflammatory signaling (Ma et al., 2024a). In metabolic disease, hyperglycemia licenses HMGB1-dependent NLRP3 activation, promoting vascular pyroptosis and BBB dysfunction, thereby linking metabolic stress to cognitive decline (Liu et al., 2022). In chemotherapy-induced peripheral neuropathy, disulfide HMGB1 released from resident macrophages—facilitated by GSDMD-dependent pyroptosis—activates neuronal TLR4 and drives NF-κB–dependent transient receptor potential vanilloid 1 (TRPV1) upregulation and mechanical allodynia; inhibiting disulfide isoform of high-mobility group box 1 (ds-HMGB1) signaling alleviates pain without compromising anticancer efficacy and correlates with clinical pain severity, supporting ds-HMGB1 as a tractable biomarker and target (Yang et al., 2025). The chemotheraphy-induced peripheral neuropathy study identifies ds-HMGB1 as a biomarker, but the finding derives from a single rodent model; human cerebrospinal fluid or plasma validation across different chemotherapy regimens is needed.

#### 3.1.4 Epilepsy

In epilepsy models, neuroinflammation is frequently associated with HMGB1/TLR4 signaling and caspase-1–linked pyroptosis readouts (Sun Y. et al., 2025). Glycyrrhizin reduces seizure burden and neuronal injury, accompanied by inhibition of the HMGB1/TLR4/NF-κB axis and downstream pyroptosis-associated signaling, suggesting that restraining HMGB1-driven inflammatory amplification may confer disease-modifying potential (Wei et al., 2025).

#### 3.1.5 Neurotoxicity

Diverse toxic exposures can converge on an HMGB1–inflammasome–pyroptosis axis in vulnerable neural populations. Prenatal sevoflurane exposure induces HMGB1 upregulation, activates TLR4/NF-κB–licensed NLRP3 signaling, and promotes caspase-1–dependent pyroptosis with persistent cognitive deficits; glycyrrhizin interrupts this cascade, implicating HMGB1 as an upstream driver of pyroptosis-linked neurodevelopmental toxicity (Shan et al., 2023).

#### 3.1.6 Neuroblastoma

HMGB1-regulated pyroptosis has also been implicated in neural-derived tumors. In neuroblastoma, HMGB1 modulates chemotherapy-induced, caspase-3/GSDME-dependent pyroptosis, thereby influencing the balance between lytic pyroptotic death and apoptosis and shaping treatment responses. HMGB1 depletion reduces GSDME cleavage and pyroptotic features in response to cisplatin or etoposide, while altering death-mode partitioning and chemosensitivity, supporting HMGB1 as a regulator of therapy-induced inflammatory death in this tumor context (Fan et al., 2023).

### 3.2 Respiratory system diseases

#### 3.2.1 Acute lung injury (ALI)/acute respiratory distress syndrome (ARDS)

ALI and its severe form ARDS are characterized by uncontrolled pulmonary inflammation, alveolar–capillary barrier disruption, and high mortality (Shen et al., 2024). Accumulating evidence supports the pyroptosis–HMGB1 axis as a principal amplifier linking inflammasome-driven lytic death to cytokine escalation and barrier dysfunction. In endotoxin-driven ALI, alveolar macrophage pyroptosis constitutes an early initiating event, with caspase-1 activation promoting IL-1β/IL-18 maturation and HMGB1 release; inhibiting caspase-1 reduces HMGB1 levels and mitigates edema and tissue injury, supporting a causal contribution of pyroptosis-associated HMGB1 output (Wu et al., 2015). HMGB1 further expands the cellular repertoire and inflammatory reach of pyroptosis by facilitating endothelial and epithelial injury. In pulmonary endothelium, HMGB1/RAGE signaling sensitizes cells to exaggerated pyroptotic responses under systemic stress, while in alveolar epithelium HMGB1 can promote cytosolic LPS delivery, enabling caspase-11–dependent noncanonical pyroptosis and amplifying tissue damage in severe inflammation (Shen et al., 2024; Yang et al., 2016). Across sterile and infectious etiologies, multiple models converge on this axis, consistent with HMGB1 functioning as both a DAMP released downstream of pyroptosis and a facilitator of inflammasome activation in neighboring compartments (Tian et al., 2021; Yang and Zhang, 2022).

Importantly, HMGB1 functions in ALI are context dependent. In specific redox settings, controlled HMGB1 preconditioning can restrain macrophage pyroptosis through antioxidant signaling, whereas in septic or ventilator-associated injury HMGB1 predominantly amplifies inflammatory escalation (Ding P. et al., 2023; Fei et al., 2020). Upstream homeostatic checkpoints such as mitophagy can therefore tune HMGB1 release and pyroptotic execution; enhancing nuclear factor erythroid 2–related factor 2 (Nrf2)-dependent mitophagy suppresses NLRP3 activation, reduces macrophage pyroptosis, and lowers HMGB1 in serum and bronchoalveolar lavage fluid, supporting mitophagy as a tractable control point over the pyroptosis–HMGB1 axis in ARDS (Liu et al., 2025).

#### 3.2.2 Chronic inflammatory airway diseases

##### 3.2.2.1 Asthma

Asthma is a heterogeneous inflammatory airway disorder in which epithelial injury can be efficiently converted into immune amplification through pyroptosis-associated pathways. In toluene diisocyanate (TDI)-induced asthma, bronchial epithelial cells exhibit caspase-1 activation, GSDMD cleavage, and extracellular release of IL-1β and HMGB1, while inhibiting NLRP3 suppresses epithelial pyroptotic signaling and attenuates airway hyperresponsiveness and inflammation (Zhuang et al., 2020). In steroid-insensitive, neutrophilic asthma, enhanced epithelial pyroptosis correlates with increased HMGB1 release and Th17-associated inflammation, and NLRP3 inhibition simultaneously reduces pyroptotic execution and Th17 polarization, supporting a mechanistic linkage between pyroptosis-derived HMGB1 and severe airway inflammation (Lin et al., 2025).

##### 3.2.2.2 Other inflammatory airway diseases

In chronic rhinosinusitis with nasal polyps, HMGB1-associated epithelial pyroptosis is coupled to tissue remodeling. LncRNA LINC01094 promotes epithelial-mesenchymal transition (EMT) and pyroptosis by upregulating HMGB1 and activating downstream remodeling pathways, suggesting that HMGB1-driven epithelial reprogramming can bridge pyroptotic signaling and structural airway pathology (Liu et al., 2024).

#### 3.2.3 Pulmonary arterial hypertension (PAH)

PAH involves progressive vascular remodeling accompanied by chronic inflammation, where inflammasome activation and pyroptosis may contribute to vascular injury. In pulmonary arterial smooth muscle cells, KIF23 has been implicated in promoting pyroptosis-associated signaling and proliferative remodeling, whereas *KIF23* knockdown attenuates vascular pathology with coordinated reductions in NLRP3 activation and HMGB1-linked inflammatory outputs, linking inflammatory fate decisions to structural progression in PAH (Wu Z. A. et al., 2022). This study uses only cultured pulmonary arterial smooth muscle cells (PASMCs) without *in vivo* animal models or patient tissue validation, so the proposed KIF23–pyroptosis–HMGB1 axis in PAH progression remains correlative at best. Whether KIF23 knockdown attenuates vascular pathology in intact animals or human PAH explants has not been tested, limiting translational relevance.

#### 3.2.4 Viral infection–associated lung injury

Viral lung injury is frequently accompanied by HMGB1 redistribution and inflammasome-related death programs that amplify tissue damage. In respiratory syncytial virus (RSV) bronchiolitis, berberine restrains HMGB1/TLR4/NF-κB signaling and reduces inflammasome activation and pyroptosis-associated execution, thereby alleviating inflammation and remodeling (Jiao and Yang, 2025). In COVID-19, SARS-CoV-2 infection engages inflammasome-linked inflammatory signaling and PANoptotic programs that promote HMGB1 release, and HMGB1 inhibition (e.g., glycyrrhizin) attenuates pyroptosis-associated injury while potentially limiting viral replication, supporting a dual anti-inflammatory and antiviral rationale for targeting HMGB1 in severe viral pneumonia (Kwak et al., 2023; Tong et al., 2022; Gowda et al., 2021).

#### 3.2.5 Lung cancer

In Non-Small Cell Lung Cancer (NSCLC), pyroptosis-associated HMGB1 release can function as either an immunogenic signal that supports antitumor immunity or a pro-tumoral mediator shaped by the tumor microenvironment (TME). Therapy-induced pyroptosis in tumor cells can elicit DAMP release including HMGB1 and promote immunogenic cell death (ICD)-like immune activation, whereas non-malignant compartments may exploit HMGB1-centered programs to sustain immune suppression (Wang J. W. et al., 2025; Hu et al., 2023). Single-cell profiling has identified an HMGB1^high tumor-associated neutrophil subset transcriptionally driven by GATA2 that engages inhibitory circuits [e.g., T cell immunoglobulin and mucin-domain containing protein 3 (TIM-3)], suggesting that HMGB1-rich myeloid niches can contribute to immune evasion and context-dependent outcomes within the TME (Hu et al., 2023). Thus, in lung cancer, the biological consequences of pyroptosis and HMGB1 are dictated by cellular compartment and immune context rather than by a uniform “beneficial” or “detrimental” model.

### 3.3 Digestive system diseases

#### 3.3.1 Liver diseases

##### 3.3.1.1 Acute liver injury (ALI)

The liver is continually exposed to metabolic stress and gut-derived inflammatory cues, rendering regulated hepatocellular death a frequent entry point across etiologies. Inflammasome-driven pyroptosis coupled to HMGB1 mobilization acts as a major amplifier of acute hepatic inflammation, although crosstalk among death programs complicates attribution to a single dominant modality (Eguchi et al., 2014; Mazzolini et al., 2016). In heatstroke, hepatic NLRP3 assembly and caspase-1 activation promote IL-1β maturation and pyroptosis, and inhibiting HMGB1 or the NLRP3–caspase-1 axis markedly attenuates inflammatory liver injury, supporting HMGB1 as an upstream driver of sterile inflammatory amplification (Geng et al., 2015). In endotoxemia and polymicrobial sepsis, HMGB1/TLR4/RAGE-linked signaling similarly associates with inflammasome activation and pyroptosis in hepatic innate immune compartments, reinforcing HMGB1 as an integrative mediator bridging systemic inflammation and acute hepatic damage (Qiu et al., 2025; Huang et al., 2020; Aboyoussef et al., 2021).

##### 3.3.1.2 Non-alcoholic liver disease (NAFLD)/non-alcoholic steatohepatitis (NASH)

In NAFLD/NASH, hepatocyte injury is driven by lipotoxicity and metabolic overload, with progression tightly coupled to sterile inflammation and fibrogenesis. While apoptosis remains prominent, inflammasome activation and pyroptosis increasingly emerge as inflammatory amplifiers that cooperate with metabolic stress pathways rather than acting as isolated death modalities (Eguchi et al., 2014; Mazzolini et al., 2016). In hepatocyte systems, endocrine regulators such as ghrelin attenuate tumor necrosis factor-α (TNF-α)–induced inflammatory injury accompanied by reduced caspase-1 activation and HMGB1 expression, highlighting convergent modulation of inflammatory death coupling under metabolic stress (Ezquerro et al., 2019). However, these *in vitro* findings lack *in vivo* validation in diet-induced NASH models, limiting translational relevance.

##### 3.3.1.3 Hepatic ischemia-reperfusion injury (HIRI)

HIRI is a clinically relevant sterile inflammatory complication of hepatectomy and transplantation. Cell-type–restricted evidence indicates that myeloid, rather than hepatocyte, GSDMD processing is a dominant driver of hepatic I/R inflammation, positioning innate immune pyroptosis upstream of tissue amplification (Li J. C. et al., 2020). Consistently, HMGB1 inhibition attenuates I/R-induced liver injury by suppressing Kupffer cell pyroptosis and downstream inflammatory escalation, supporting HMGB1-centered pyroptosis as a tractable therapeutic target in sterile hepatic injury (Hua et al., 2019). Nevertheless, whether HMGB1 acts upstream of GSDMD or merely as a downstream DAMP remains unclear, as isoform-specific or compartment-specific approaches have not been applied.

##### 3.3.1.4 Hepatic fibrosis (HF)

Fibrosis reflects persistent injury and chronic inflammation, with hepatic stellate cell activation as a central effector event. Pyroptosis can contribute upstream by supplying HMGB1 and inflammatory cues that promote stellate activation. Hepatocyte pyroptosis–associated HMGB1 release enhances HSC proliferation and migration, and inhibiting inflammasome/caspase-1 signaling mitigates this inflammatory relay, supporting an HMGB1-dependent mechanism linking lytic hepatocyte injury to fibrogenic remodeling (Wu J. W. et al., 2022). These findings are currently limited to *in vitro* systems using hepatocyte cell lines and conditioned media. Future studies should employ *in vivo* lineage-tracing or conditional knockout to determine whether hepatocyte-derived HMGB1 drives fibrosis in intact animals, along with functional validation of HMGB1 and pyroptosis markers in human fibrotic liver samples.

##### 3.3.1.5 Acute-on-chronic liver failure (ACLF)

ACLF is characterized by acute decompensation with systemic inflammation and high mortality. Experimental models support a feed-forward circuit in which HMGB1 promotes hepatocyte pyroptosis and cytokine escalation, and HMGB1 inhibition reduces caspase-1 activation and ameliorates injury (Hou et al., 2021). In hepatitis B–associated ACLF, activated NK cells induce GSDMD-dependent hepatocyte pyroptosis, triggering robust HMGB1 release that promotes NET formation and inflammatory escalation, illustrating an intercellular amplification loop linking cytotoxicity to systemic inflammation (Zhao Q. et al., 2025). Critically, whether HMGB1 neutralization improves survival in ACLF has not been tested in large animal models or clinical trials.

##### 3.3.1.6 Hepatocellular carcinoma (HCC)

In HCC, pyroptosis-related pathways exert stage- and context-dependent effects. After insufficient radiofrequency ablation, GSDME-associated pyroptosis has been linked to immunosuppressive remodeling and reduced responsiveness to PD-L1 blockade, indicating that pyroptotic programs can be co-opted to shape unfavorable immune contexts (Liang X. X. et al., 2024). Single-cell analyses further suggest macrophage-centered inflammatory niches with coordinated pyroptosis-associated mediator expression, including HMGB1-linked signaling, reinforcing the view that pyroptosis–HMGB1 crosstalk in HCC is largely governed by immune–tumor interactions within the microenvironment (Luo et al., 2025).

#### 3.3.2 Esophageal cancer (EC)

In esophageal cancer, transcriptomic analyses consistently indicate dysregulated pyroptosis-related gene programs that stratify prognosis and associate with immune infiltration and inferred immunotherapy responsiveness, implicating pyroptosis-associated inflammatory states in shaping the tumor immune landscape (Jiang et al., 2023; Zhang et al., 2022). Functional evidence further suggests therapeutic inducibility: irradiation can trigger caspase-3/GSDME-mediated pyroptosis accompanied by release of ICD-associated DAMPs including HMGB1, and genetic disruption of caspase-3 or GSDME abrogates these inflammatory death and DAMP signals, supporting the concept that therapy-driven pyroptosis may be harnessed to promote antitumor immunity in EC (Tan et al., 2023).

#### 3.3.3 Pancreatic diseases

Acute pancreatitis exemplifies a hyperinflammatory disorder in which pyroptosis bridges immune activation and parenchymal injury. In hyperlipidemic AP, palmitic acid–activated macrophages promote acinar pyroptosis via an HMGB1-dependent TLR4/NLRP3 axis, and baicalein alleviates pancreatic injury by disrupting HMGB1 engagement with TLR4/NLRP3 and reducing inflammasome activation and GSDMD cleavage (Wang X. Y. et al., 2025). Targeting the execution step can also confer protection: disulfiram inhibits GSDMD-dependent membrane permeabilization, reduces cytokine output and tissue injury, and alleviates systemic inflammatory complications in severe AP models, supporting pyroptosis as a mechanistic driver of both local damage and systemic amplification (Zhao T. M. et al., 2025).

#### 3.3.4 Gastric cancer (GC)

In reflux-associated gastric carcinogenesis, macrophage inflammasome activation can serve as an upstream inflammatory engine. USP50 deubiquitinates ASC, amplifying NLRP3 inflammasome activation and pyroptosis-associated HMGB1 release; macrophage-derived HMGB1 then activates proliferative signaling in gastric epithelium via the phosphoinositide 3-kinase/protein kinase B (PI3K/AKT) and mitogen-activated protein kinase/extracellular signal-regulated kinase (MAPK/ERK) pathways, promoting tumor progression and positioning the pyroptosis–HMGB1 axis as a bridge between chronic inflammation and epithelial transformation (Zhao et al., 2024).

#### 3.3.5 Colorectal diseases

##### 3.3.5.1 Colitis

In colitis, excessive epithelial inflammatory death accelerates barrier failure and amplifies mucosal inflammation. Lipocalin-2 (LCN2), enriched in ulcerative colitis epithelium, exacerbates dextran sulfate sodium (DSS) colitis by activating the NF-κB/NLRP3/GSDMD axis, promoting intestinal epithelial cells (IECs) pyroptosis and HMGB1 release; genetic deletion or silencing of LCN2 attenuates epithelial injury and inflammatory mediator production, establishing epithelial pyroptosis as a causal effector of mucosal damage (Yang Y. Y. et al., 2024).

##### 3.3.5.2 Colorectal cancer (CRC)/colitis-associated cancer (CAC)

Pyroptosis exerts compartment- and context-dependent effects in colorectal tumorigenesis. In sporadic CRC under metabolic stress, caspase-6–driven activation of GSDMC family members induces tumor pyroptosis and HMGB1 release, which upregulates chemokine attractant C-X-C motif chemokine 2 (CXCL2) and recruits myeloid-derived suppressor cells (MDSCs) via CXCL2–C-X-C chemokine receptor type 2 (CXCR2) signaling, thereby establishing an immunosuppressive circuit that accelerates progression (Gao H. C. et al., 2025). Thus, tumor cell pyroptosis can function as an immunoregulatory “switch,” favoring immune evasion when coupled to MDSC recruitment. Conversely, therapeutically induced pyroptosis can be leveraged to enhance antitumor immunity: engineered oncolytic adenoviruses induce tumor pyroptosis with HMGB1 release, activate TLR4–MyD88–NF-κB–NLRP3 signaling in tumor-associated macrophages, promote M1 polarization, and enhance cytotoxic T-cell infiltration, supporting HMGB1 as a productive immune-activating signal when pyroptosis is engaged in an immunostimulatory context (Wang S. et al., 2025).

##### 3.3.5.3 Hirschsprung-associated enterocolitis (HAEC)

In HAEC, HMGB1 released within a pyroptosis-enriched inflammatory milieu promotes macrophage extracellular trap formation via TLR4-dependent p38 MAPK and NF-κB activation, exacerbating oxidative stress and epithelial injury and reinforcing a self-amplifying inflammatory loop (Zhang R. et al., 2025).

#### 3.3.6 Intestinal injury

Across diverse stressors, intestinal barrier failure is frequently driven by inflammasome activation, epithelial pyroptosis, and DAMP release. In sepsis models, carbon monoxide donors preserve barrier integrity and reduce mortality while suppressing both canonical and noncanonical pyroptotic signaling and lowering inflammatory mediators including HMGB1, supporting epithelial pyroptosis as a key effector of sepsis-associated gut injury (Wang H. Z. et al., 2020). In radiation injury, caspase-3–dependent GSDME cleavage contributes to epithelial pyroptosis with HMGB1 release, and antioxidant or cytoprotective interventions mitigate both pyroptotic and apoptotic epithelial loss (Shi et al., 2023). In neonatal necrotizing enterocolitis, LCN2 has been implicated as an upstream driver of NLRP3/caspase-1/GSDMD activation and HMGB1 release, and its inhibition attenuates intestinal injury and improves survival, positioning pyroptosis-associated HMGB1 output as a mechanistic node in developmental barrier vulnerability (Chen et al., 2026).

### 3.4 Circulatory system diseases

In the circulatory system, HMGB1 frequently sits at the interface between sterile danger signaling (e.g., metabolic stress, oxidative injury, and crystal deposition) and inflammasome-driven pyroptosis, thereby amplifying vascular inflammation and tissue injury. Through this positioning, HMGB1 also bridges pyroptosis with other regulated death programs and repair responses, rendering it a context-dependent driver and an attractive therapeutic node in cardiovascular pathology (Shi et al., 2025).

#### 3.4.1 Atherosclerotic vascular disease

##### 3.4.1.1 Atherosclerosis and coronary atherosclerotic complications

Atherosclerosis is sustained by chronic sterile inflammation in which DAMP–PRR signaling licenses inflammasome activation and culminates in pyroptotic membrane rupture. This lytic execution increases extracellular HMGB1 and amplifies inflammatory signaling, thereby accelerating plaque progression and destabilization (Truong et al., 2021; Xu X. D. et al., 2022). Consistent with this framework, pharmacologic blockade of pyroptosis execution provides lesion-level protection: inhibition of caspase-1 or GSDMD reduces plaque burden, macrophage infiltration, endothelial activation markers (vascular cell adhesion molecule-1 [VCAM-1], monocyte chemoattractant protein-1 [MCP-1]), and aortic levels of cleaved GSDMD, IL-1β, and HMGB1 in ApoE−/− mice, with concordant suppression of macrophage pyroptosis *in vitro* (Zhang B. L. et al., 2023).

Upstream regulatory mechanisms further shape HMGB1-centered pyroptotic amplification. In a sepsis-exacerbated coronary atherosclerosis model, macrophage CCAAT/enhancer-binding protein beta (CEBPB) upregulation enhanced HMGB1 transcription and pyroptosis, increased reactive oxygen species (ROS) production, and drove endothelial VCAM-1 induction via NF-κB signaling. Disruption of this CEBPB/HMGB1/VCAM-1 axis mitigated endothelial dysfunction and attenuated lesion progression, illustrating how systemic inflammation accelerates coronary disease through HMGB1-dependent pyroptotic signaling (Zhang S. Y. et al., 2025). In parallel, macrophage-derived extracellular vesicles delivering siHMGB1 suppressed caspase-11–dependent pyroptosis, reduced foam-cell formation, and delayed plaque development in ApoE^−/−^ mice, highlighting an EV-mediated HMGB1–caspase-11 circuit that modulates inflammatory plaque biology (Liang W. J. et al., 2024).

##### 3.4.1.2 Vascular calcification and coronary artery calcification (CAC)

Vascular calcification represents an advanced atherosclerotic remodeling phenotype rather than a fully independent disease entity. In experimental CAC models, a caspase-3/GSDME axis links apoptotic stress to pyroptosis-like membrane rupture and HMGB1 release in vascular smooth muscle cells (VSMCs). Genetic or pharmacologic interference with GSDME or HMGB1 reduced inflammatory recruitment, macrophage infiltration, and calcific deposition, whereas hematopoietic GSDME knockdown provided limited benefit, implicating VSMCs as a dominant source and target of this pathway during CAC progression (Yang H. H. et al., 2024). Epitranscriptomic regulation may further tune gasdermin execution: bone marrow stromal cell–derived exosomes enriched in alkB homolog 5 (ALKBH5), an m6A RNA demethylase, attenuated VSMC calcification and pyroptosis-associated phenotypes by modulating m6A-dependent GSDME expression, thereby limiting HMGB1 release (Xu et al., 2025).

##### 3.4.1.3 Endothelial dysfunction

Endothelial dysfunction represents an early functional entry point into atherogenic disease. In acute hypercholesterolemia, endothelial NLRP3 activation increased caspase-1 activity and HMGB1 expression and directly impaired endothelium-dependent vasodilation. Genetic deletion of *Nlrp3* or pharmacologic inhibition of caspase-1 or HMGB1 restored vasoreactivity, and recombinant HMGB1 was sufficient to induce dysfunction in otherwise normal arteries, supporting HMGB1 as an active effector of endothelial injury rather than a passive byproduct of inflammation (Zhang et al., 2015). Metabolic stress converges on similar mechanisms: in hyperhomocysteinemia, cathepsin V–dependent lysosomal destabilization enabled caspase-1/GSDMD activation and HMGB1 release, linking metabolic injury to endothelial pyroptosis and vascular inflammation (Leng et al., 2020).

##### 3.4.1.4 Abdominal aortic aneurysms (AAA)

In AAA, macrophage–VSMC communication may transmit pyroptotic competence via exosome-associated non-coding RNAs. Exosomes derived from M1 macrophages were enriched in lncRNA PVT1, which promoted Ang II–induced VSMC inflammation and pyroptosis by functioning as a ceRNA for miR-186-5p and derepressing HMGB1. Silencing exosomal PVT1 reduced inflammasome activation, GSDMD cleavage, and inflammatory cytokine production, supporting a PVT1/miR-186-5p/HMGB1 axis linking inflammatory macrophages to pyroptosis-driven vascular wall degeneration (Zhang et al., 2024).

#### 3.4.2 Inflammatory vasculopathies and vasculitis

Beyond classical autoimmune vasculitides, endotoxemia and sepsis can manifest as fulminant inflammatory vasculopathy dominated by endothelial pyroptosis. Endothelial GSDMD emerges as a decisive determinant of vascular injury and lethality: endothelial-specific Gsdmd deletion—rather than myeloid Gsdmd ablation—conferred robust protection against barrier disruption and mortality. Mechanistically, hepatocyte-derived HMGB1 released in a GSDMD-dependent manner engaged endothelial RAGE signaling, amplifying endothelial pyroptosis and systemic vascular injury. Pharmacologic inhibition of endothelial GSDMD alleviated vascular damage and improved survival, establishing an endothelial GSDMD–HMGB1–RAGE axis as a central driver of sepsis-associated inflammatory vasculopathy (Su et al., 2025). Similar HMGB1/RAGE–NLRP3–GSDMD signaling has been reported in Kawasaki disease–associated coronary vasculitis, suggesting a shared HMGB1-centered pyroptotic axis in acute inflammatory vasculopathies (Jia et al., 2019).

#### 3.4.3 Cardiac diseases

##### 3.4.3.1 Myocardial ischemia–reperfusion injury (MIRI)

Multiple lines of evidence place HMGB1 upstream of inflammasome activation and gasdermin execution during myocardial I/R (Ding H. S. et al., 2023). Non-anticoagulant heparin protected cardiomyocytes from hypoxia/reoxygenation injury by binding HMGB1 and disrupting HMGB1–RAGE engagement, thereby inhibiting caspase-11/GSDMD activation and oxidative stress (Wang X. W. et al., 2025). At the level of stress adaptation, HMGB1 also intersected with redox signaling: hypoxia promoted HMGB1 redistribution and suppressed the Nrf2/heme oxygenase-1 (HO-1) antioxidant program, whereas HMGB1 knockdown reduced NLRP3/caspase-1/GSDMD activation and attenuated myocardial injury (Zheng F. Z. et al., 2025). Beyond cardiomyocyte-intrinsic mechanisms, endothelial pyroptosis independently contributed to microcirculatory dysfunction after reperfusion. Endothelial cell–specific HMGB1 deletion preserved barrier integrity, reduced infarct size, and suppressed AIM2 inflammasome activation, supporting an endothelial HMGB1–AIM2 pathway as an additional driver of reperfusion injury (Chen R. et al., 2025).

##### 3.4.3.2 Drug-induced cardiomyopathy/systemic inflammatory cardiotoxicity

In doxorubicin cardiotoxicity, inflammation-driven pyroptosis contributes to cardiomyocyte injury. MSC-derived exosomes reduced HMGB1/TLR4 signaling, NLRP3 inflammasome assembly, and downstream pyroptosis markers (caspase-1 activation, IL-1β/IL-18 release, GSDMD cleavage) in doxorubicin (DOX)-treated cardiomyocytes, suggesting that paracrine/exosomal therapy can suppress HMGB1-linked inflammasome activation as part of cardioprotection (Ali and Singla, 2024). In sepsis-induced myocardial dysfunction, exosomes from apelin-pretreated MSCs provided superior functional rescue relative to baseline MSC exosomes and reduced cardiomyocyte pyroptosis. Mechanistically, exosomal miR-34a-5p targeted HMGB1 and engaged the HMGB1/AMP-activated protein kinase (AMPK) axis, and depletion of miR-34a-5p partially abrogated benefit—supporting exosome payload optimization as a tractable strategy to modulate HMGB1 and pyroptosis in inflammatory cardiomyopathy (Li T. et al., 2025).

#### 3.4.4 Hematologic malignancies

Although not classically categorized as cardiovascular disease, hematologic malignancies directly interface with circulatory physiology and systemic inflammatory homeostasis. In multiple myeloma, a MYC G-quadruplex (G4) stabilizer induced inflammasome-associated pyroptosis characterized by caspase-1 activation, GSDMD cleavage, HMGB1 nuclear-to-cytoplasmic translocation, and ballooning morphology, suggesting that therapeutically induced pyroptosis may reshape tumor–immune inflammatory circuits in blood cancers (Gaikwad et al., 2020; Leu et al., 2021).

### 3.5 Urinary system diseases

#### 3.5.1 Renal tubular dysfunction

Renal tubular pyroptosis can be driven by pathogen-associated cues as well as gut–kidney metabolic crosstalk that modulates HMGB1 availability and lysosomal stability. In an LPS-induced sepsis-associated AKI model, oral administration of cardamonin (CAD) for 1 week preserved renal function and attenuated tubular injury, oxidative stress, inflammation, and pyroptosis. Notably, CAD was undetectable in serum but accumulated in the colon, where it remodeled the local metabolome and markedly increased columbianetin acetate (CA). Oral administration of CA reproduced the renoprotective effects of CAD, and integrative network pharmacology, molecular docking, and surface plasmon resonance analyses identified HMGB1 as a principal binding target. Mechanistically, CA competitively bound HMGB1, antagonized LPS–HMGB1 interactions, reduced cellular LPS uptake, and stabilized lysosomes, thereby restraining tubular inflammasome activation upstream of gasdermin-mediated membrane rupture. Collectively, these findings support a gut-derived metabolite–HMGB1 competitive-binding mechanism that disrupts LPS-driven tubular pyroptosis at an early inflammatory licensing stage (Zhou Z. H. et al., 2025).

#### 3.5.2 Acute kidney injury (AKI)

In systemic inflammatory states, renal pyroptosis is tightly coupled to macrophage activation and cytokine–DAMP amplification loops. In a combined acute liver failure and AKI model induced by LPS/D-galactosamine, blockade of TNF-α signaling attenuated the TNF-α–HMGB1 axis and reduced tubular pyroptosis both *in vivo* and *in vitro*. These effects were accompanied by reduced M1 macrophage infiltration and improved liver and kidney function, whereas macrophage polarization toward a pro-inflammatory phenotype amplified TNF-α/HMGB1 signaling and pyroptotic injury. Pharmacological inhibition of HMGB1 similarly restrained tubular pyroptosis, supporting a TNF-α–HMGB1 pathway that links myeloid activation to renal injury during systemic inflammation (Wang Y. et al., 2020).

Chemotherapy-associated nephrotoxicity provides a distinct but clinically relevant context in which oxidative stress and innate immune signaling converge on pyroptotic execution. In cisplatin-induced AKI, suppression of NF-κB signaling attenuated NLRP3 inflammasome activation, reduced HMGB1 expression, and limited caspase-1 activation, GSDMD cleavage, and IL-1β/IL-18 maturation. These findings underscore that cisplatin nephrotoxicity reflects intertwined apoptotic and pyroptotic programs, with HMGB1 acting as a central inflammatory amplifier downstream of NF-κB–licensed inflammasome activation (Li C. J. et al., 2025).

#### 3.5.3 Ischemia–reperfusion (I/R)–induced kidney injury

Renal ischemia–reperfusion injury creates a permissive environment for death-program crosstalk driven by oxidative stress and DAMP release. Beyond canonical inflammasome activation, environmental toxicants highlight sequential coupling between distinct death modalities. In a neonicotinoid (imidacloprid)-induced nephrotoxicity model, ferroptosis emerged as an initiating injury event characterized by lipid peroxidation and impaired Nrf2-dependent antioxidant responses. Inhibition of ferroptosis attenuated subsequent NLRP3-driven inflammation, reduced macrophage accumulation in proximal tubules, and suppressed activation of the HMGB1–RAGE/TLR4–NF-κB axis. These observations support a hierarchical injury model in which ferroptotic stress promotes HMGB1-centered inflammatory signaling that subsequently potentiates pyroptosis and sustains renal dysfunction (Zhang D. F. et al., 2023).

#### 3.5.4 Chronic kidney disease (CKD)

Progression of chronic kidney disease reflects a transition from acute epithelial injury to persistent inflammation and fibrosis, processes in which tubular pyroptosis plays a decisive role. In obstructive nephropathy, metabolic stress favored a caspase-3/GSDME–dependent pyroptotic fate in renal tubular cells, marked by generation of the GSDME-N fragment. Genetic deletion of *Casp3* or Gsdme alleviated tubular injury, inflammation, and fibrotic remodeling, whereas hematopoietic contributions were comparatively limited. Mechanistically, HMGB1 released from pyroptotic tubular cells amplified myeloid inflammasome activation and fibrogenic signaling, while tubular-specific *Hmgb1* deletion attenuated macrophage caspase-11 activation and IL-1β production. These findings identify tubular HMGB1 as a parenchymal-derived amplifier linking epithelial pyroptosis to chronic inflammatory scarring (Li Y. S. et al., 2021).

Metabolic dysregulation can similarly sustain CKD progression through HMGB1-centered pathways. In hypercholesterolemic models, suppression of oxidized low-density lipoprotein (Ox-LDL)–associated HMGB1 signaling attenuated PI3K/mechanistic target of rapamycin (mTOR) activation, NLRP3 inflammasome execution, and renal inflammation, suggesting that under lipid-driven stress, HMGB1 functions upstream of nutrient-sensing and pyroptotic programs to promote chronic kidney injury (Elnagar et al., 2022). Toxicological models further reinforce this concept: oxidative stress–induced lysosomal destabilization activated a caspase-3/GSDME–HMGB1 axis, linking apoptotic–pyroptotic switching to inflammatory amplification during chronic renal injury (Ma et al., 2024b).

#### 3.5.5 Bladder cancer

In urothelial malignancies, therapeutically induced pyroptosis can be leveraged to convert local tumor injury into systemic immune activation. A microwave-sensitized nanotherapeutic platform loaded with the DNA methylation inhibitor decitabine induced robust GSDME-dependent pyroptosis, enhanced tumor immunogenicity, and potentiated responses to anti–PD-1 therapy *in vivo*. These findings support a “pyroptosis programming” paradigm in bladder cancer, in which epigenetic priming and physical sensitization converge on gasdermin-mediated immunogenic cell death to remodel the tumor immune microenvironment and enhance immunotherapeutic efficacy (Deng et al., 2024).

### 3.6 Locomotor system diseases

#### 3.6.1 Osteoarthritis (OA) and knee osteoarthritis (KOA)

Osteoarthritis, particularly knee osteoarthritis (KOA), is increasingly recognized as a chronic inflammatory disorder in which synovial inflammation and fibrosis contribute to pain, structural decline, and joint dysfunction. Evidence indicates that pyroptosis in synovial macrophages and fibroblast-like synoviocytes (FLSs) fuels KOA inflammation, with HMGB1 acting as a key DAMP linking lytic death to stromal remodeling. In experimental KOA, depletion of synovial macrophages or inhibition of caspase-1 attenuates inflammasome activation (NLRP3/ASC/GSDMD), decreases IL-1β/IL-18 and HMGB1 release, and reduces synovitis-associated fibrosis and tissue injury, supporting macrophage pyroptosis as an upstream initiator of inflammatory–fibrotic cascades (Zhang et al., 2019). Mechanistically, HMGB1 released from pyroptotic macrophages functions as a paracrine cue that activates synovial fibroblasts through the HMGB1/RAGE/TGF-β1/mothers against decapentaplegic homolog 3 (SMAD3) axis; blocking HMGB1, RAGE, or SMAD3 alleviates synovial fibrosis and joint pathology *in vivo*, identifying HMGB1 as a molecular bridge between innate immune cell death and stromal remodeling (Wu et al., 2023). In addition to macrophages, inflammasome-dependent pyroptosis can occur in FLSs, and genetic or pharmacological blockade of inflammasome components reduces HMGB1 release and mitigates joint damage, consistent with a self-reinforcing loop within the synovial microenvironment.

Cartilage cells may also participate in this network, although their contribution appears more context dependent. *In vitro*, LPS/ATP-induced chondrocyte injury is accompanied by increased pyroptosis-associated signals and HMGB1 elevation, and inhibition of GSDMD (e.g., disulfiram) or HMGB1 (e.g., glycyrrhizic acid) partially restores viability and dampens inflammatory outputs. Notably, combined blockade of GSDMD and HMGB1 aggravated oxidative stress in this model, suggesting that over-suppression of inflammatory death pathways may have unintended redox consequences and emphasizing the need for balanced intervention strategies (Li C. et al., 2021). Upstream regulatory layers further converge on HMGB1-centered pyroptosis. enhancer of zeste homolog 2 (EZH2)-mediated repression of miR-142-3p relieves inhibition of HMGB1 expression, thereby enhancing ER stress–associated chondrocyte pyroptotic signaling and accelerating cartilage degeneration (Chen Y. et al., 2025). In parallel, broader miRNA networks modulate NLRP3/caspase-1 activation and HMGB1 release, positioning miRNAs as upstream regulators of the pyroptosis–HMGB1 axis and potential biomarkers in KOA (An et al., 2023). Therapeutic studies further reinforce the centrality of HMGB1: chrysin alleviated macrophage pyroptosis and neuropathic pain in KOA by suppressing HMGB1-regulated RAGE/PI3K/AKT signaling, with HMGB1 overexpression reversing its protective effects (Xu et al., 2024). Together, these findings support pyroptosis-derived HMGB1 as a context-dependent hub that integrates synovial immune activation, stromal remodeling, and downstream cartilage deterioration in OA/KOA.

#### 3.6.2 Bone, muscle, and spine disorders

Beyond joint pathology, HMGB1-associated pyroptotic programs contribute to musculoskeletal degeneration and maladaptive repair in bone and mechanically stressed tissues. In osteoporosis, lncRNA SNHG1 interacts with HMGB1 to promote caspase-1/GSDMD activation and IL-1β/IL-18 release in bone marrow mesenchymal stem cells, thereby impairing osteogenic differentiation; inhibition of HMGB1 or caspase-1 partially restores osteoblastogenesis, indicating an HMGB1–pyroptosis axis that links inflammatory death signaling to impaired bone formation (Pan et al., 2024). In trauma-induced heterotopic ossification, macrophage pyroptosis promotes aberrant osteogenesis through HMGB1-containing extracellular vesicles. Transfer of HMGB1 to tendon-derived stem cells activates TLR9–NF-κB signaling and senescence programs, and blockade of macrophage pyroptosis reduces ectopic bone formation, highlighting pyroptosis-derived HMGB1 as a mediator of maladaptive regeneration (Li J. H. et al., 2023).

Skeletal muscle degeneration also displays involvement of the pyroptosis–HMGB1 axis. In chronic obstructive pulmonary disease (COPD)-associated muscle atrophy, cigarette smoke exposure increases circulating and intramuscular HMGB1 and activates HMGB1/TLR4–NLRP3–caspase-1–GSDMD signaling, while HMGB1 inhibition alleviates inflammatory wasting phenotypes, supporting a systemic link between chronic inflammatory states and muscle decline (Xiong et al., 2025). Intervertebral disc degeneration (IVDD) provides a related example in a mechanically stressed tissue where metabolic cues converge on inflammatory death pathways. Obesity-associated stress activates NLRP3 signaling in nucleus pulposus cells through a mitochondria-derived double-stranded RNA-activated protein kinase R (PKR) pathway, promoting HMGB1 release; metformin reduces mitochondrial damage, suppresses HMGB1 release, and alleviates disc degeneration (Gao S. et al., 2025). Rosuvastatin similarly inhibits TNF-α-induced pyroptosis-associated signals, senescence, and matrix catabolism by downregulating HMGB1/NF-κB signaling, reinforcing HMGB1 as a convergence point for inflammatory and metabolic injury in IVDD (Chen et al., 2023).

### 3.7 Endocrine system diseases

Diabetes mellitus is a prototypical endocrine–metabolic disorder characterized by persistent hyperglycemia, dyslipidemia, oxidative stress, and chronic low-grade inflammation, collectively creating a permissive milieu for inflammasome activation and inflammatory cell injury across tissues. Emerging evidence supports pyroptosis as a recurrent injury module in diabetes complications, with HMGB1 amplifying downstream inflammation and fibrosis. In diabetic skeletal muscle, metabolic overload is associated with increased pyroptosis-related markers and elevated HMGB1, accompanied by fibrosis and sarcopenia, and bone morphogenetic protein-7 (BMP-7) reduces lipid deposition, inflammatory infiltration, HMGB1 expression, and pyroptosis-associated readouts while preserving muscle architecture and function (Narasimhulu and Singla, 2023). In diabetic kidney disease (DKD), podocyte injury provides a clinically central example: high-glucose stress triggers ROS accumulation, NLRP3 activation, and HMGB1 release, while diosgenin attenuates oxidative stress and pyroptosis by engaging Nrf2-dependent antioxidant defenses and suppressing NLRP3 signaling, thereby preserving podocyte viability (Tang et al., 2024). Across tissues, these studies converge on a stress–inflammasome–pyroptosis module in which HMGB1 functions as a key amplifier of inflammatory deterioration and fibrotic remodeling, supporting therapeutic strategies that target upstream inflammasome licensing, redox imbalance, or HMGB1-mediated signaling.

### 3.8 Reproductive system diseases

#### 3.8.1 Female reproductive system diseases

Female reproductive diseases illustrate the dual nature of the pyroptosis–HMGB1 axis: pathogenic amplification in inflammatory and pregnancy-related disorders, yet therapeutically inducible immunogenic death in gynecologic malignancies. In breast cancer, clinically used neoadjuvant chemotherapy regimens (paclitaxel and anthracyclines) can induce caspase-9/caspase-3–GSDME cleavage accompanied by HMGB1 and ATP release, thereby enhancing phagocytic clearance and cytokine production by immune cells—features consistent with pyroptosis-coupled ICD that may contribute to heterogeneous pathological complete response outcomes (Fu et al., 2024). Beyond conventional chemotherapy, a carrier-free chemophotodynamic nanoplatform co-assembled from cytarabine and chlorin e6 (Ce6) induces ROS-dependent, GSDME-mediated pyroptosis together with canonical ICD hallmarks (HMGB1/ATP release and calcitonin exposure), resulting in suppression of primary and recurrent tumors and improved responsiveness to anti–PD-1 therapy *in vivo* (Li L. et al., 2023). These findings highlight HMGB1 as a principal DAMP linking tumor-cell pyroptosis to immune activation in breast cancer.

In ovarian cancer, endogenous regulators of terminal membrane rupture may influence progression and treatment resistance. NINJ1 expression decreases from early to advanced disease and low NINJ1 levels correlate with worse overall survival, while cisplatin-resistant ovarian cancer cells exhibit reduced NINJ1 expression compared with cisplatin-sensitive counterparts, suggesting that impaired terminal membrane rupture, and potentially reduced DAMP release (e.g., HMGB1), may contribute to tumor evolution and drug resistance (Berkel and Cacan, 2023). Conversely, pyroptosis can be pharmacologically induced to enhance antitumor immunity: the polo-like kinase 1 (PLK1) inhibitor BI 2536 triggers caspase-3/GSDME-dependent pyroptosis and HMGB1 release, accompanied by increased intratumoral CD8^+^ T-cell accumulation (Huo et al., 2022). Together, these data indicate that ovarian cancer biology may be shaped by both intrinsic rupture competence and therapeutically inducible pyroptosis pathways that position HMGB1 at the interface between tumor injury and immune modulation.

Chronic inflammation is a key driver of cervical epithelial injury and carcinogenic progression, but evidence in this setting often derives from expression readouts rather than direct demonstration of membrane rupture. In LPS-stimulated cervical epithelial cells, HMGB1/RAGE signaling promotes inflammatory cytokine production and invasive phenotypes, accompanied by upregulation of pyroptosis-associated markers such as NLRP3 and caspase-4; inhibition of RAGE attenuates these responses (You et al., 2021). Post-transcriptional regulation provides additional restraint, as miR-129-5p restoration suppresses HMGB1/RAGE signaling and reduces caspase-1 activation and GSDMD-N generation under inflammatory stress (Wa et al., 2025).

Inflammatory uterine disorders provide more direct support for HMGB1-driven pyroptosis in shaping local immune imbalance. In chronic endometritis, elevated HMGB1 is accompanied by increased GSDMD-N-terminal (NT) signals and macrophage association, and glycyrrhizic acid suppresses pyroptosis-associated inflammation *in vivo* (Yang et al., 2023). Endometriosis similarly involves inflammasome activation with concurrent HMGB1 upregulation in ectopic lesions, consistent with a pyroptosis-associated inflammatory milieu that supports invasive behavior (Huang et al., 2022). Pregnancy complications such as preeclampsia and unexplained recurrent spontaneous abortion are likewise characterized by excessive sterile inflammation at the maternal–fetal interface, where placental stressors converge on inflammasome–gasdermin signaling and alarmin release (including HMGB1), amplifying IL-1β/IL-18–skewed inflammation and disrupting immune tolerance (Banerjee et al., 2021; Zhou Y. et al., 2025). In unexplained recurrent spontaneous abortion (URSA) models, low-dose aspirin attenuates HMGB1 signaling and pyroptosis markers, consistent with interruption of a macrophage–HMGB1–TLR–inflammasome loop that promotes pregnancy loss (Zhu et al., 2021).

#### 3.8.2 Male reproductive system diseases

In prostate cancer, pyroptosis can act as a tumor-suppressive program that is mechanistically coupled to HMGB1 release. Zinc finger DHHC-type containing 1 (ZDHHC1) is downregulated in prostate cancer and low expression correlates with poor prognosis. ZDHHC1 overexpression increases secretion of IL-1β, IL-18, LDH, and HMGB1 and suppresses tumor cell viability, while mechanistic analyses indicate that ZDHHC1 palmitoylates GSDMD to facilitate GSDMD-N membrane translocation and execution of pyroptosis; inhibition of palmitoylation or pyroptosis partially reverses these effects (You et al., 2025). Beyond malignancy, inflammatory prostate disorders also involve HMGB1-centered amplification. In experimental autoimmune prostatitis, IL-17A-driven epithelial injury is linked to extracellular release of disulfide HMGB1, which promotes macrophage M1 polarization through a Janus kinase 2/signal transducer and activator of transcription 1–phosphofructokinase, platelet type (JAK2/STAT1–PFKP) glycolytic axis; inhibition of HMGB1 or Stat1 attenuates macrophage activation and inflammation (Ma et al., 2025).

Environmental toxicants and anticancer therapies further illustrate death-program crosstalk in male reproductive injury, with HMGB1 serving as a convergent inflammatory mediator. In Leydig cells, chlormequat chloride triggers apoptosis, pyroptosis, and ferroptosis with robust inflammatory responses, and ferroptosis inhibition more effectively rescues cell viability and reduces inflammatory mediator release (including HMGB1) than pan-caspase blockade, suggesting ferroptosis-initiated stress as a dominant upstream event with downstream HMGB1 amplification (Wang et al., 2024). In doxorubicin-induced testicular injury, olmesartan restores testicular function and suppresses HMGB1/NLRP3/GSDMD signaling while enhancing autophagy and antioxidant defenses, supporting coordinated modulation of pyroptosis-associated inflammation and cytoprotective pathways in chemotoxic injury (Elariny et al., 2025).

Although these preclinical findings are promising, they largely rely on cancer cell lines, acute toxicant exposure models, or rodent immunization systems without human tissue validation or *in vivo* genetic confirmation of HMGB1’s causal role.

### 3.9 Inflammatory and autoimmune diseases

#### 3.9.1 Infection-associated inflammatory diseases

##### 3.9.1.1 Infection and sepsis

Sepsis is a prototypical dysregulated host response to infection in which inflammasome activation, pyroptosis, and DAMP release drive systemic inflammation and multi-organ dysfunction. HMGB1 is widely recognized as a late-phase mediator in sepsis that integrates regulated cell-death programs (including pyroptosis) with DAMP-driven inflammatory amplification (Liang and He, 2022; Zhang et al., 2020). Mechanistically, HMGB1 functions as an endotoxin-delivery protein by binding extracellular LPS and facilitating its cytosolic entry through RAGE-dependent uptake and lysosomal destabilization. This process licenses noncanonical inflammasome activation, leading to caspase-11 cleavage, GSDMD pore formation, pyroptosis, and lethality in endotoxemia and bacterial sepsis (Den et al., 2018). These insights have motivated interaction-targeted strategies that disrupt HMGB1-dependent endotoxin delivery and downstream noncanonical pyroptosis. Glycyrrhizin competitively binds HMGB1, interferes with HMGB1–LPS interactions, and attenuates caspase-11–associated inflammation, organ injury, and lethality. Heparin derivatives, independent of anticoagulant activity, can similarly block HMGB1–LPS binding and prevent cytosolic LPS delivery and caspase-11 activation, providing a mechanistic basis for immunomodulatory benefit (Tang et al., 2021). An 8-hydroxyquinoline derivative (NSC84094) directly targets HMGB1, disrupts HMGB1–LPS binding, suppresses caspase-11 signaling, and protects against endotoxemic organ injury and death (Wang et al., 2021). In pneumonia-associated sepsis, IL-17 pathway activation correlates with increased inflammasome activation, cleaved caspase-1 and GSDMD, elevated LDH, and enhanced IL-1β and IL-18 release. Concurrent upregulation of HMGB1 and RAGE, together with IL-17A–tumor necrosis factor receptor–associated factor 6 (TRAF6)–NF-κB signaling, supports an immune axis linking Th17-skewed inflammation to pyroptotic amplification in pulmonary infection–associated sepsis (Li L. L. et al., 2020).

##### 3.9.1.2 Sterile trauma and systemic inflammatory response syndrome (SIRS)

Unlike infection-triggered sepsis, sterile mechanical trauma induces an early and massive HMGB1 surge that involves active inflammasome- and gasdermin-mediated pyroptosis, not passive necrosis. A landmark clinical study by Peltz and colleagues systematically profiled plasma HMGB1 kinetics in severely injured trauma patients, revealing a rapid and marked elevation within the first hour after injury, with levels continuing to rise and peaking within several hours—a kinetic pattern that contrasts sharply with the late and modest HMGB1 increase observed in sepsis. Further analysis ruled out transfused blood products as a major source, confirming that endogenous host tissue release predominates. Although peak HMGB1 levels did not correlate with traditional prognostic scores, patients requiring prolonged intensive care consistently exhibited higher HMGB1 levels early after injury. These findings position HMGB1 as an early pathogenic mediator in post-traumatic SIRS and multiple organ dysfunction syndrome, supporting the therapeutic potential of targeting HMGB1 or its upstream pyroptotic machinery in sterile inflammatory conditions such as severe trauma, hemorrhagic shock, and ischemia-reperfusion injury (Peltz et al., 2009).

##### 3.9.1.3 Bacterial toxins and hyperinflammatory states

Certain bacterial toxins directly engage inflammasome pathways to induce pyroptosis and DAMP release. Cytolethal distending toxin activates caspase-1 and caspase-4, promotes GSDMD cleavage, and induces pyroptosis with HMGB1 release in human macrophages via glycogen synthase kinase 3β (GSK3β)–spleen tyrosine kinase (Syk) signaling, identifying toxin-proximal kinases as regulators of inflammasome execution (Shenker et al., 2020). In toxin-driven systemic inflammation, anthrax lethal toxin elicits rapid NLRP1-dependent IL-1β release *in vivo* and is associated with increased circulating HMGB1 and NET-related mediators, suggesting that DAMP amplification can accompany early cytokine storm states (Greaney et al., 2020).

#### 3.9.2 Autoimmune and immune dysregulation disorders

In allergic rhinitis, inflammasome activation is associated with HMGB1 redistribution and mucosal inflammation. AIM2 inflammasome components (AIM2, ASC, and caspase-1 p20) increase following allergen challenge, whereas NLRP3 alterations are less pronounced, suggesting an AIM2-biased inflammasome response. The concomitant epithelial injury pattern without canonical apoptotic cleavage signatures is compatible with pyroptosis-like epithelial damage coupled to HMGB1 signaling (Wang Y. et al., 2022).

#### 3.9.3 Skin and mucocutaneous inflammatory syndromes

Drug-induced mucocutaneous toxicity can involve caspase-3/GSDME-dependent pyroptosis, which couples epithelial injury to HMGB1 release and local inflammation. epidermal growth factor receptor tyrosine kinase inhibitors (EGFR-TKIs) such as afatinib induce caspase-3/GSDME cleavage in keratinocytes and sebocytes, accompanied by HMGB1 and IL-1α release, and inhibition of caspase-3 or knockdown of GSDME suppresses these responses, supporting GSDME cleavage as a mechanistic basis for EGFR-TKI–associated skin toxicities (Zhu et al., 2025). In ischemic skin injury, paeoniflorin improves distal flap survival and angiogenesis while reducing HMGB1 levels and dampening TLR4/NF-κB signaling together with inflammasome-associated readouts, including NLRP3, caspase-1, IL-1β, and IL-18 (Li et al., 2022). In dermatitis-like inflammation, extracellular vesicles derived from Ecklonia cava suppress TLR4/NF-κB/NLRP3 signaling, reduce GSDMD-NT and cytokine maturation, and decrease HMGB1, supporting vesicle-based approaches to restrain pyroptosis-linked inflammatory amplification in skin (Kim et al., 2024).

**FIGURE 2:**
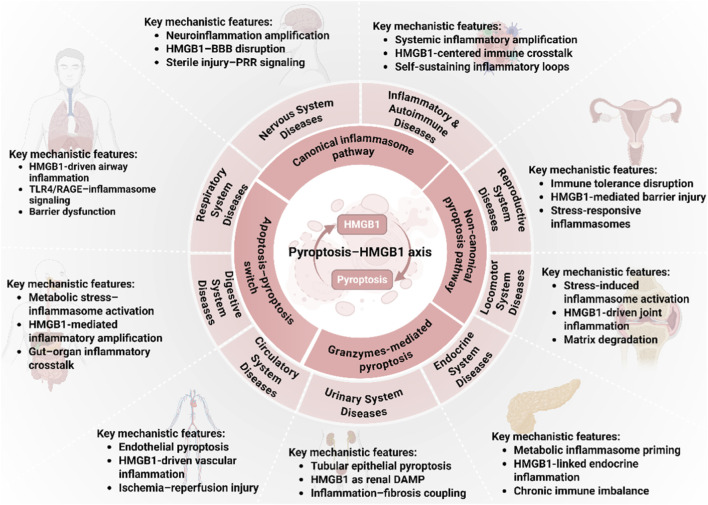
The pyroptosis–HMGB1 axis across systemic diseases. The pyroptosis–HMGB1 axis functions as a convergent inflammatory module across diverse organ systems, linking inflammasome- and gasdermin-driven membrane rupture to damage-associated molecular pattern (DAMP) amplification, immune remodeling, and secondary tissue injury. Despite disease-specific upstream triggers, including infection, metabolic stress, ischemia–reperfusion, toxins, and sterile inflammation, recurrent regulatory themes emerge across systems, notably pattern recognition receptor (PRR) signaling via TLR4 and RAGE, inflammasome platforms such as NLRP3 and AIM2, and execution checkpoints involving caspase-1/4/5/11 and gasdermins. These shared pathways shape cytokine release, barrier dysfunction, and self-sustaining inflammatory loops in the nervous, respiratory, digestive, circulatory, urinary, endocrine, locomotor, reproductive, and immune systems. The schematic highlights how context-dependent engagement of canonical and non-canonical pyroptosis pathways underpins systemic inflammation and organ dysfunction, providing a framework for cross-disease comparison and therapeutic targeting.

In summary, the pyroptosis–HMGB1 axis represents a convergent inflammatory module across diverse systemic diseases, coupling inflammasome/gasdermin-driven membrane rupture to DAMP amplification and immune remodeling. Although the upstream triggers vary by tissue and disease context (e.g., infection, metabolic stress, ischemia–reperfusion, toxins, or sterile inflammation), several regulatory themes recur, including PRR signaling (TLR4/RAGE), inflammasome platforms (notably NLRP3 and AIM2), and execution checkpoints (caspase-1/4/5/11 and GSDMD/GSDME) that collectively shape cytokine release, barrier dysfunction, and secondary injury cascades. Therapeutically, an expanding repertoire of interventions—spanning natural compounds, small-molecule inhibitors, biologics, and EV-based delivery strategies—has demonstrated potential to attenuate pathological pyroptosis and/or reprogram HMGB1 signaling toward disease-modifying outcomes, while in selected cancer settings, controlled pyroptosis can be leveraged to promote immunogenic danger signaling and enhance antitumor immunity. To facilitate cross-disease comparison and underscore recurring mechanistic and translational themes, Table 1 summarizes representative diseases, dominant pathways, HMGB1-related roles, and potential therapeutic targets. To support cross-disease comparison and mechanistic integration, Figure 2 provides an integrated schematic of the pyroptosis–HMGB1 circuitry underpinning systemic inflammation and organ dysfunction.

**TABLE 1:** Representative roles of the pyroptosis–HMGB1 axis across systemic diseases.

Disease	Key pathway	Role of pyroptosis-HMGB1	Therapeutic target/Strategy	Ref.
Nervous system				
Acute CNS injury (ICH, I/R, TBI, SCI/SCI-R)	HMGB1-TLR4-NLRP3s; NET-NINJ1 feed-forward loop	HMGB1 amplifies neuroinflammation and BBB disruption via canonical pyroptosis (caspase-1/GSDMD)	HMGB1 inhibition, TLR4 blockade, NLRP3 suppression, NINJ1 targeting	Lei et al. (2024), Yao and Li (2023), Zhang et al. (2025a), Liang et al. (2021), Zheng et al. (2025a), Guo et al. (2022)
Chronic neuroinflammatory/neurodegenerative disorders	HMGB1/RAGE/NLRP3 signaling	HMGB1 sustains chronic neuroinflammation through persistent pyroptotic signaling	HMGB1-inflammasome signaling suppression	Homma et al. (2024), Yang et al. (2020), Chen et al. (2022b), Saad et al. (2025)
Sepsis/metabolic stress-associated neurological complications	EV/miRNA-mediated HMGB1 suppression; hyperglycemia-NLRP3 signaling	HMGB1 drives pain via neuronal GSDMD-dependent pyroptosis	HMGB1 blockade, TLR4 inhibition	Ma et al. (2024a), Liu et al. (2022), Yang et al. (2025)
Respiratory system				
ALI/ARDS	HMGB1-facilitated cytosolic LPS delivery	HMGB1 drives cytokine storm and barrier injury through macrophage/endothelial pyroptosis	Caspase-1 inhibition, HMGB1 neutralization	Shen et al. (2024), Wu et al. (2015), Yang et al. (2016), Tian et al. (2021), Yang and Zhang (2022), Ding et al. (2023a), Fei et al. (2020), Liu et al. (2025)
Asthma/chronic airway inflammation	NLRP3/GSDMD, pyroptosis	HMGB1 converts epithelial injury to Th17 inflammation via epithelial pyroptosis	NLRP3 inhibition, HMGB1/TLR4/NF-κB suppression	Zhuang et al. (2020) Lin et al. (2025)
Viral lung injury/COVID-19	HMGB1/TLR4/NF-κB; PANoptosis	HMGB1 amplifies hyperinflammation through inflammasome-linked pyroptosis	HMGB1-targeting anti-inflammatory approaches	Jiao and Yang (2025), Kwak et al. (2023), Tong et al. (2022), Gowda et al. (2021)
Lung cancer (NSCLC)	GSDME (therapy-induced); GATA2/HMGB1/TIM-3	Context-dependent: HMGB1 can be immunogenic (tumor pyroptosis) or immunosuppressive (myeloid niches)	Pyroptosis; HMGB1	Wang et al. (2025a), Hu et al. (2023)
Digestive system				
Acute liver injury/HIRI/ACLF	HMGB1-TLR4/RAGE; myeloid GSDMD	HMGB1 drives sterile/septic injury via Kupffer cell pyroptosis	HMGB1 inhibition, NLRP3/caspase-1 blockade	Zhao et al. (2025a), Geng et al. (2015), Qiu et al. (2025), Huang et al. (2020), Aboyoussef et al. (2021), Li et al. (2020a), Hua et al. (2019), Hou et al. (2021)
NAFLD/NASH/HF	Metabolic stress-inflammasome-HMGB1	HMGB1 links lipotoxicity to inflammation/fibrosis through hepatocyte/immune pyroptosis	inflammasome/caspase-1 inhibition, HMGB1 suppression	Eguchi et al. (2014), Mazzolini et al. (2016), Ezquerro et al. (2019), Wu et al. (2022b)
HCC/EC/GC	Context-dependent (GSDME, ASC/NLRP3)	HMGB1 has dual roles: immunosuppressive or ICD-promoting, via tumor or macrophage pyroptosis	HMGB1 binding; GSDMD inhibition	Liang et al. (2024a), Luo et al. (2025), Jiang et al. (2023), Zhang et al. (2022), Tan et al. (2023), Zhao et al. (2024)
Pancreatitis/intestinal injury	HMGB1-TLR4-NLRP3 signaling	HMGB1 drives local and systemic inflammation via macrophage-acinar pyroptosis	HMGB1 neutralization	Wang et al. (2025b), Zhao et al. (2025b), Wang et al. (2020a), Shi et al. (2023), Chen et al. (2026)
CRC/CAC	HMGB1-CXCL2-CXCR2	HMGB1 can be immunosuppressive (MDSC recruitment) or immunogenic depending on pyroptosis context	Oncolytic viruses; immune modulation	Gao et al. (2025a), Wang et al. (2025c), Zhang et al. (2025b)
Circulatory system				
Atherosclerosis/vascular disease	HMGB1-VCAM1; PI3K/AKT; CEBPB-HMGB1 axis	HMGB1 drives endothelial dysfunction and plaque progression via macrophage/endothelial pyroptosis	Caspase-1/GSDMD inhibition; HMGB1 blockade	Truong et al. (2021), Xu et al. (2022a), Zhang et al. (2023a), Zhang et al. (2025c), Liang et al. (2024b)
Vascular calcification	Caspase-3-GSDME axis; ALKBH5/m6A regulation	HMGB1 links apoptosis to inflammatory calcification via VSMC pyroptosis-like rupture	GSDME/HMGB1 inhibition	Yang et al. (2024a), Xu et al. (2025)
Inflammatory vasculopathy/vasculitis	Endothelial GSDMD-HMGB1-RAGE; NLRP3 signaling	HMGB1 causes systemic vascular leakage via endothelial pyroptosis	Endothelial GSDMD inhibition; HMGB1-RAGE blockade	Su et al. (2025), Jia et al. (2019)
MIRI	HMGB1-RAGE; NLRP3; AIM2	HMGB1 promotes cardiac inflammation and oxidative stress through cardiomyocyte/endothelial pyroptosis	HMGB1 inhibition	Ding et al. (2023b), Wang et al. (2025d), Zheng et al. (2025b), Chen et al. (2025a)
Cardiotoxicity	HMGB1-TLR4-NLRP3; HMGB1-AMPK	HMGB1 mediates cardiomyocyte injury via pyroptosis	GSDMD/HMGB1 blockade	Ali and Singla (2024), Li et al. (2025a)
Urinary system				
AKI/renal I/R injury	HMGB1-LPS interaction; TNF-α-HMGB1 axis	HMGB1 amplifies tubular injury and macrophage activation via tubular pyroptosis (caspase-1/GSDMD or GSDME)	HMGB1 inhibition	Wang et al. (2020b), Li et al. (2025b), Zhang et al. (2023b)
CKD	Metabolic stress-HMGB1; caspase-3-GSDME	HMGB1 drives chronic inflammation–fibrosis coupling through tubular pyroptosis	Inflammasome/HMGB1 targeting	Li et al. (2021a), Elnagar et al. (2022), Ma et al. (2024b)
Bladder cancer	Epigenetic priming-GSDME; HMGB1-ICD	HMGB1 enhances tumor immunogenicity via GSDME-mediated pyroptosis	Pyroptosis programming	Deng et al. (2024)
Locomotor system				
OA/KOA	HMGB1/RAGE/TGF-β1/SMAD3	HMGB1 links pyroptosis to synovial fibrosis and pain via macrophage/FLS/chondrocyte pyroptosis	HMGB1, RAGE, SMAD3, or inflammasome blockade	Zhang et al. (2019), Wu et al. (2023), Li et al. (2021b), Chen et al. (2025b), An et al. (2023), Xu et al. (2024), Pan et al. (2024), Li et al. (2023a), Xiong et al. (2025), Gao et al. (2025b), Chen et al. (2023)
Endocrine system				
Diabetes/DKD	Hyperglycemia/ROS-NLRP3–HMGB1 signaling	HMGB1 amplifies inflammation, fibrosis, and podocyte injury through pyroptosis	NLRP3/HMGB1 inhibition	Narasimhulu and Singla (2023), Tang et al. (2024)
Reproductive system				
Reproductive cancers (breast, ovarian, prostate)	Caspase-3/GSDME; HMGB1-DAMP signaling; PLK1-GSDME	HMGB1 has dual role: ICD (beneficial) or progression depending on tumor pyroptosis context	ICD induction	Berkel and Cacan (2023), Huo et al. (2022), You et al. (2025)
endometritis, endometriosis, prostatitis	HMGB1-RAGE/TLR4	HMGB1 drives local inflammation and immune imbalance via epithelial/macrophage pyroptosis	HMGB1 inhibition	You et al. (2021), Wa et al. (2025), Yang et al. (2023), Huang et al. (2022)
preeclampsia, URSA	Inflammasome-gasdermin- HMGB1	HMGB1 disrupts maternal–fetal immune tolerance through placental/immune pyroptosis	HMGB1 suppression HMGB1 suppression	Banerjee et al. (2021), Zhou et al. (2025b), Zhu et al. (2021)
Testicular injury/toxicant-induced damage	HMGB1-NLRP3/GSDMD; ferroptosis-pyroptosis crosstalk	HMGB1 mediates inflammatory amplification via Leydig cell pyroptosis	HMGB1/NLRP3 inhibition	Wang et al. (2024), Elariny et al. (2025)
Inflammatory and autoimmune diseases				
Sepsis/infection-associated inflammation	HMGB1-LPS delivery-caspase-11-GSDMD	HMGB1 acts as late mediator integrating DAMP amplification via noncanonical pyroptosis	HMGB1-LPS interaction blockade	Liang and He (2022), Zhang et al. (2020), Den et al. (2018), Tang et al. (2021), Wang et al. (2021), Li et al. (2020b)
Sterile trauma/SIRS	HMGB1 surge via inflammasome/gasdermin-mediated pyroptosis	HMGB1 acts as early pathogenic driver of post-traumatic SIRS and MODS	HMGB1 inhibition, targeting pyroptotic machinery	Peltz et al. (2009)
Bacterial toxin-induced hyperinflammation	GSK3β-Syk; NLRP1/NLRP3 inflammasomes	Amplifies early cytokine storm and DAMP release via pyroptosis	inflammasome inhibition	Shenker et al. (2020), Greaney et al. (2020)
Allergic rhinitis	AIM2 inflammasome; HMGB1 redistribution	HMGB1 promotes epithelial injury and immune dysregulation through pyroptosis-like damage	Inflammasome modulation; HMGB1 pathway inhibition	Wang et al. (2022a)
Skin and mucocutaneous inflammatory syndromes	Caspase-3-GSDME; TLR4/NF-κB/NLRP3	HMGB1couples epithelial injury to local inflammation via keratinocyte/sebocyte pyroptosis	Caspase-3/GSDME targeting	Zhu et al. (2025), Li et al. (2022), Kim et al. (2024)

## 4 Therapeutic perspectives targeting pyroptosis-HMGB1 axis

Throughout this review, HMGB1 is primarily presented as a pro-inflammatory DAMP. However, emerging evidence reveals a context-dependent duality. In sterile and infectious inflammation, the disulfide isoform of HMGB1 activates TLR4/RAGE to promote NLRP3 inflammasome assembly and pyroptosis, and its neutralization protects against organ damage. In contrast, within the tumor microenvironment, HMGB1 exerts immunosuppressive effects by inducing myeloid-derived suppressor cells, polarizing macrophages toward M2, upregulating PD-L1, and promoting regulatory T cells. During chemotherapy- or radiotherapy-induced immunogenic cell death (ICD), HMGB1 released from dying tumor cells acts as an immune adjuvant that enhances antitumor immunity—opposite to its chronic immunosuppressive role. This paradox is resolved by recognizing that the functional outcome depends on HMGB1’s redox state, concentration, cellular source, and receptor utilization. The pyroptosis–HMGB1 axis itself exhibits duality: pyroptotic HMGB1 release worsens inflammatory organ damage but, when therapeutically induced in cancer, can stimulate antitumor immunity. Thus, HMGB1 is a context-dependent rheostat, and recognizing this duality is essential for precision therapies. Given this duality, multiple therapeutic strategies have been developed to modulate the pyroptosis–HMGB1 axis according to disease context. These approaches can be classified into four major categories: (i) direct HMGB1 neutralization; (ii) modulation of inflammasomes, caspases, or gasdermins; (iii) nanomedicine- and biomaterial-enabled delivery; and (iv) multi-target regimens including traditional Chinese medicine (TCM). Representative interventions are summarized in Table 2.

**TABLE 2:** Therapeutic strategies targeting the pyroptosis–HMGB1 axis across systemic diseases.

Agent	Primary targets	Disease models	Mechanistic actions on pyroptosis–HMGB1 axis	Major outcomes	Ref.
HMGB1 neutralization/sequestration					
Glycyrrhizin	HMGB1–LPS interaction; HMGB1-mediated cytosolic LPS delivery	Sepsis, endotoxemia; ALI; hepatic I/R; NEC-associated brain injury; epilepsy; SARS-CoV-2 models	Competitively binds HMGB1, blocks HMGB1–LPS interaction, inhibits cytosolic LPS delivery and non-canonical caspase-11/4 activation, suppressing downstream GSDMD-mediated pyroptosis	Reduced cytokine storm, organ injury, mortality; neuroprotection; antiviral-associated inflammation control	Wei et al. (2025), Gowda et al. (2021), Hua et al. (2019), Wang et al. (2022b), Yao and Sun (2019), Sun et al. (2025b)
Heparin/non-anticoagulant heparinoids	Extracellular HMGB1; HMGB1–LPS complexes; endothelial glycocalyx	Inflammatory tissue injury; sepsis-associated vascular and inflammatory complications	Sequesters extracellular HMGB1 and HMGB1–LPS complexes, preserves macrophage glycocalyx integrity, thereby preventing cytosolic LPS delivery and caspase-11-dependent pyroptosis, independent of anticoagulant activity	Attenuated inflammatory injury with translational feasibility	Wang et al. (2025d)
Anti-HMGB1 antibodies/peptides	Extracellular HMGB1	LPS-stimulated macrophages	Neutralizes extracellular HMGB1, suppressing HMGB1-triggered and LPS-associated macrophage pyroptosis	Reduced caspase-1 activation and pyroptotic cell death in macrophages	Shang et al. (2023)
Inflammasome modulation					
MCC950	NLRP3 inflammasome	Systemic inflammation (tool compounds)	Blocks canonical NLRP3–caspase-1–GSDMD pyroptosis; ATP-induced caspase-3/GSDME-mediated pyroptosis and HMGB1 release may persist	Partial suppression of pyroptosis; HMGB1 release and alternative caspase-3/GSDME pathways may persist	Zeng et al. (2019)
Gasdermin-targeted strategies					
Disulfiram	GSDMD pore formation	Endotoxin shock; systemic inflammation	Covalent inhibition of GSDMD pore formation	Protection from lethal endotoxemia	Zhao et al. (2025b)
Dimethyl fumarate (DMF)	GSDMD cysteine residues	Inflammatory injury models; pyroptosis-associated cellular injury	Electrophile-mediated inhibition of gasdermin pores	Reduced inflammatory cell death and tissue injury	Pei et al. (2025)
Anti-GSDME single-domain antibody (sdAb)	GSDME-NT	Chemotherapy-induced lung injury	Neutralizes GSDME-mediated pyroptosis and HMGB1 release	Reduced systemic toxicity and HMGB1 release without compromising anticancer efficacy	Ma et al. (2023)
Nanomedicine and biomaterial platforms (anti-inflammatory)					
Apelin-MSC-derived exosomes	miR-34a-5p/HMGB1–AMPK	Sepsis-induced myocardial dysfunction	miRNA-mediated suppression of HMGB1 and cardiomyocyte pyroptosis	Improved cardiac function and survival	Li et al. (2025a)
hucMSC-derived exosomes	Caspase-11/4	DSS colitis	Inhibits non-canonical macrophage pyroptosis via miRNA cargo	Inhibits caspase-11/4–driven non-canonical macrophage pyroptosis via miRNA cargo	Xu et al. (2022b)
EVs from *Ecklonia cava*	TLR4/NF-κB/NLRP3; HMGB1	Skin inflammation	Suppresses inflammasome activation and HMGB1 release	Attenuated dermatitis-like inflammation	Kim et al. (2024)
Nanomedicine and biomaterial platforms (pro-pyroptotic/ICD)					
Carrier-free chemo-PDT nanoplatform (Ara-C/Ce6)	Caspase-3/GSDME	Breast cancer	Induces controlled pyroptosis and HMGB1/ATP release, promotes ICD	Enhanced antitumor immunity and abscopal effects	Li et al. (2023b)
Ir(III)–lonidamine conjugates	Mitochondria; caspase-3/GSDME	Cervical cancer	Mitochondrial ROS → GSDME-dependent pyroptosis with HMGB1 release	ICD induction and tumor suppression	Lu et al. (2025)
ER-targeted Ir(III) photosensitizer	ER stress; GSDME	Breast cancer	Photo-activated pyroptosis with robust HMGB1/ATP release	DC maturation and systemic antitumor immunity	Zhi et al. (2024)
Ag/Au bimetallic nanoparticles	Multiple death pathways	Solid tumor cell lines	Context-dependent induction of pyroptosis/necroptosis and HMGB1 release	Modulation of DAMP profiles	Katifelis et al. (2022)
Traditional Chinese medicine/multi-target agents					
Berberine	HMGB1/TLR4/NF-κB	RSV-induced bronchiolitis	Suppresses pyroptosis, EMT, fibrosis and HMGB1 signaling	Reduced viral inflammation and fibrosis	Jiao and Yang (2025)
Baicalein	HMGB1/TLR4/NLRP3	Hyperlipidemic acute pancreatitis	Direct HMGB1 binding; inhibits macrophage M1 polarization and acinar pyroptosis	Alleviated pancreatic injury	Wang et al. (2025b)
Bletilla striata polysaccharides	NLRP3/caspase-1/GSDMD; HMGB1	ARDS	Suppresses alveolar macrophage pyroptosis and cytokine storm via NLRP3/caspase-1/GSDMD and HMGB1/TLR4 pathways	Improved survival and lung injury	Wu et al. (2024)
Diosgenin	Nrf2/NLRP3; HMGB1	Diabetic kidney disease	Antioxidant-driven suppression of inflammasome and pyroptosis	Podocyte protection	Tang et al. (2024)
Genistein	Caspase-1/GSDMD; HMGB1	Macrophage inflammation	Prevents K^+^ efflux and ROS-dependent caspase-1/GSDMD-mediated pyroptotic lysis with reduced HMGB1 release	Reduced cytokine and HMGB1 release	Yang and Zhang (2024)
Paeoniflorin	HMGB1/TLR4/NLRP3	Ischemic skin flap	Inhibits pyroptosis while promoting angiogenesis	Improved flap survival	Li et al. (2022)
Columbianetin acetate	HMGB1–LPS binding	Sepsis-associated AKI	Competes with LPS for HMGB1 binding, suppresses tubular pyroptosis	Renal protection	Zhou et al. (2025a)

ALI, acute lung injury; AKI, acute kidney injury; AMPK, AMP-activated protein kinase; ARDS, acute respiratory distress syndrome; ASC, apoptosis-associated speck-like protein containing a caspase recruitment domain; ATP, adenosine triphosphate; DAMP, damage-associated molecular pattern; DC, dendritic cell; DMF, dimethyl fumarate; DSS, dextran sulfate sodium; ER, endoplasmic reticulum; EV, extracellular vesicle; FGF21, fibroblast growth factor 21; GSDMD, gasdermin D; GSDME, gasdermin E; GSDME-NT, gasdermin E N-terminal fragment; HMGB1, high mobility group box 1; ICD, immunogenic cell death; I/R, ischemia–reperfusion; LPS, lipopolysaccharide; MCC950, NLRP3 inflammasome inhibitor MCC950; MSC, mesenchymal stem cell; NEC, necrotizing enterocolitis; NF-κB, nuclear factor kappa-B; NLRP3, NOD-like receptor family pyrin domain-containing 3; ROS, reactive oxygen species; RSV, respiratory syncytial virus; sdAb, single-domain antibody; TCM, traditional Chinese medicine; TLR4, toll-like receptor 4.

### 4.1 Mechanism-oriented modulation of the pyroptosis–HMGB1 axis

#### 4.1.1 Targeting HMGB1 release, translocation, or extracellular activity

A direct and mechanistically intuitive strategy for attenuating pyroptosis-associated pathology is to limit extracellular HMGB1 availability or disrupt HMGB1–receptor interactions. Glycyrrhizin, a clinically used natural compound, represents a prototypical HMGB1-binding agent with broad preclinical validation across inflammatory and infectious disease models. In sepsis and endotoxemia, glycyrrhizin competitively binds HMGB1 and interferes with HMGB1-facilitated cytosolic delivery of LPS, thereby attenuating downstream inflammatory cascades and systemic injury (Wang Z. T. et al., 2022). Consistent with this mechanism, glycyrrhizin reduces HMGB1 accumulation together with pyroptosis-associated readouts in multiple organ injury settings, including bacterial infection–induced acute lung injury (Yao and Sun, 2019), hepatic ischemia–reperfusion injury (via dampening HMGB1-dependent gasdermin execution in hepatic macrophages) (Hua et al., 2019), and neuroinflammatory injury in necrotizing enterocolitis models (Sun Q. et al., 2025). Beyond inflammation control, glycyrrhizin also exhibits antiviral-relevant effects by reducing HMGB1 release and suppressing viral replication in cell-based SARS-CoV-2 studies, supporting its potential repositioning value in infection-associated hyperinflammation (Gowda et al., 2021).

In parallel, heparin and non-anticoagulant heparin derivatives have emerged as therapeutically attractive HMGB1-targeting modalities, particularly in settings where anticoagulation risk must be minimized. These agents function primarily as HMGB1-sequestering or neutralizing compounds and have shown efficacy in inflammatory tissue injury and vascular complications. Their translational appeal lies in the availability of clinically advanced heparinoids and the feasibility of engineering reduced-anticoagulant derivatives that retain anti-inflammatory HMGB1-binding capacity (Wang X. W. et al., 2025).

Additional HMGB1-directed strategies include neutralizing antibodies and HMGB1-binding peptides designed to block extracellular HMGB1 bioactivity and interrupt downstream receptor signaling. Experimental evidence indicates that extracellular HMGB1 can act upstream of macrophage pyroptosis, and antibody-mediated HMGB1 neutralization suppresses LPS-associated pyroptotic responses (Shang et al., 2023). While such biologics offer high target specificity, practical considerations—including tissue penetration, dosing frequency, and immune complex–related effects—will need careful evaluation in translational development. Conditional knockout studies have shown that myeloid-specific *Hmgb1* deletion abrogates NLRP3 inflammasome activation and GSDMD cleavage in sepsis and ischemia–reperfusion injury, while endothelial-specific deletion attenuates myocardial reperfusion injury. Additionally, EV-encapsulated HMGB1 resists degradation, signals via TLR4/RAGE, and contributes to immunosuppression in cancer, plaque progression in atherosclerosis, and systemic inflammation in sepsis, complementing pharmacological findings and reinforcing HMGB1 as a non-redundant target.

#### 4.1.2 Modulating upstream inflammasomes and gasdermin execution

Given that pyroptosis is executed by inflammatory caspases and gasdermin pore formation, an alternative therapeutic strategy is to intervene at the level of inflammasomes, caspase activation, or gasdermin execution, thereby reducing inflammatory cell lysis and limiting DAMP and cytokine release. Among inflammasome-centered approaches, NLRP3 inhibitors have been widely employed as experimental tools and increasingly explored for therapeutic development. However, compensatory or bypass mechanisms may limit their efficacy. For example, when canonical NLRP3 signaling is pharmacologically inhibited, ATP can still trigger caspase-3/GSDME-dependent pyroptosis accompanied by HMGB1 release (Zeng et al., 2019). This observation underscores the importance of pathway-aware stratification (e.g., canonical versus alternative gasdermin usage) and suggests that effective clinical suppression of pathogenic pyroptosis may require combined targeting of upstream sensing and downstream execution.

At the execution level, gasdermin inhibition has progressed from small-molecule electrophiles to biologics. Disulfiram directly inhibits GSDMD pore formation via covalent modification of a critical cysteine residue and protects mice from lethal endotoxin challenge, supporting its repurposing potential in systemic hyperinflammatory conditions (Zhao T. M. et al., 2025). Dimethyl fumarate (DMF) similarly limits gasdermin pore formation and inflammatory cell death, further supporting the feasibility of electrophile-based gasdermin targeting at the execution stage of pyroptosis (Pei et al., 2025). Beyond small molecules, innovative biologics such as single-domain antibodies (sdAbs) against the GSDME N-terminal fragment have been engineered to mitigate chemotherapy-induced pyroptosis, reducing systemic HMGB1 release and lung injury *in vivo*. These studies provide proof-of-concept that selective gasdermin neutralization can preserve therapeutic efficacy while limiting inflammatory toxicity (Ma et al., 2023).

Upstream inhibition of caspase-1 and caspase-11 (human caspase-4/5) remains therapeutically relevant, particularly in infection and sepsis, where uncontrolled inflammatory caspase activation drives systemic injury. In addition to direct enzymatic inhibition, strategies that disrupt HMGB1-mediated endotoxin delivery, such as glycyrrhizin, can indirectly attenuate non-canonical caspase activation, offering a mechanistically coherent combination approach (Wang Z. T. et al., 2022). Endogenous anti-inflammatory modulators, including metabolic hormones such as fibroblast growth factor 21 (FGF21) discussed above, may further serve as physiological “brakes” on inflammasome activity, potentially improving safety profiles compared with complete pathway blockade (Ding P. et al., 2023).

### 4.2 Nanomedicine and biomaterial-based strategies

Nanomedicine and biomaterial platforms offer unique advantages for targeting the pyroptosis–HMGB1 axis, including cell- or tissue-selective delivery, dose sparing, and spatiotemporal control of inflammatory modulation or ICD. In inflammatory disease contexts, EV–based therapies have shown particular promise. MSC–derived exosomes, including engineered or preconditioned variants, can attenuate organ dysfunction by suppressing pyroptosis and modulating HMGB1-associated signaling networks. For example, Apelin-pretreated MSC exosomes deliver miRNA cargo that suppresses cardiomyocyte pyroptosis and improves cardiac function in sepsis-induced myocardial dysfunction, with HMGB1 identified as a key regulatory node (Li T. et al., 2025). Similarly, human umbilical cord MSC–derived exosomes alleviate experimental colitis by suppressing caspase-11/4-driven macrophage pyroptosis through miRNA-mediated regulation (Xu Y. T. et al., 2022). These studies support the concept of EVs as “biological nanoparticles” capable of reprogramming inflammasome and pyroptosis circuits while minimizing systemic immunosuppression.

In oncology, nanomedicine platforms have been actively developed to induce controlled pyroptosis and ICD to enhance antitumor immunity. A representative example is a carrier-free chemo-photodynamic nanoplatform that triggers GSDME-mediated pyroptosis and releases DAMPs, including HMGB1 and ATP, thereby promoting dendritic cell maturation and systemic antitumor immune responses in breast cancer models (Li L. et al., 2023). Complementarily, photoactivatable metal-complex systems enable spatial confinement of pyroptosis induction to illuminated tumors. Cyclometalated iridium(III)–lonidamine conjugates localize to mitochondria and induce caspase-3/GSDME-dependent pyroptosis with robust DAMP release (Lu et al., 2025), while endoplasmic reticulum–targeted iridium(III) photosensitizers enhance tumor immunotherapy by inducing pyroptosis and HMGB1/ATP release upon light activation (Zhi et al., 2024). Collectively, these examples highlight a clinically attractive design principle, localized activation coupled with immune amplification, which reduces systemic toxicity while leveraging pyroptosis-driven immunogenicity (Chen R. C. et al., 2025; Singh et al., 2024).

More broadly, nanomaterials themselves can influence death-pathway selection and DAMP release profiles. Bimetallic nanoparticles, such as Ag/Au alloys, can trigger mixed programmed death programs and modulate extracellular HMGB1 release in a cell-context-dependent manner (Katifelis et al., 2022). While these findings expand the therapeutic toolbox for immunogenic tumor killing, they also emphasize the need for careful materials optimization to avoid excessive inflammation in non-tumor tissues.

### 4.3 Traditional Chinese medicine and multi-target regulation

TCM-derived monomers and formulations provide a complementary therapeutic avenue through coordinated regulation of multiple pathological processes, often combining anti-inflammatory, antioxidative, barrier-protective, and immunomodulatory effects. Within this category, several compounds and herbal extracts have been reported to attenuate pyroptosis-associated injury while concurrently suppressing HMGB1 signaling. Berberine alleviates RSV-induced pediatric bronchiolitis and fibrosis by suppressing the HMGB1/TLR4/NF-κB axis and reducing both canonical and non-canonical pyroptosis markers *in vitro* and *in vivo* (Jiao and Yang, 2025). Bletilla striata polysaccharides protect against LPS-induced ARDS by inhibiting alveolar macrophage pyroptosis and attenuating both NLRP3/caspase-1/GSDMD and HMGB1/TLR4 signaling pathways (Wu et al., 2024). In hyperlipidemic acute pancreatitis, baicalein directly engages HMGB1 and mitigates macrophage polarization and acinar cell pyroptosis through HMGB1/TLR4/NLRP3-associated networks (Wang X. Y. et al., 2025). Additional examples include artemisinin analog SM934, which improves epithelial barrier dysfunction in experimental colitis with reductions in pyroptosis and HMGB1-related inflammatory markers (Shao et al., 2022), and other phytochemicals such as genistein that suppress caspase-1/GSDMD-dependent pyroptotic lysis and inflammatory mediator release (Yang and Zhang, 2024).

The principal advantage of multi-target strategies lies in their ability to address the networked nature of pyroptosis–HMGB1 biology, encompassing oxidative stress, barrier dysfunction, immune-cell polarization, and tissue remodeling. However, this breadth also introduces challenges related to standardization, target attribution, and pharmacokinetics across disease contexts. Mechanism-informed biomarkers—such as HMGB1 redox states or subcellular compartments, gasdermin cleavage signatures, and DAMP release dynamics—may facilitate rational integration of TCM candidates into modern drug development pipelines and combination strategies. Therapeutic modulation of the pyroptosis–HMGB1 axis must ultimately be guided by disease context. In sepsis and immune-mediated tissue injury, excessive pyroptosis amplifies cytokine storms and DAMP release; inhibition at the level of HMGB1, caspases, or gasdermins is therefore protective (Zhao T. M. et al., 2025; Wang Z. T. et al., 2022; Pei et al., 2025). In contrast, in cancer, controlled and localized induction of pyroptosis may enhance immunogenicity through DAMP release, antigen presentation, and T cell priming (Li L. et al., 2023; Zhi et al., 2024; Chen R. C. et al., 2025; Singh et al., 2024). This duality also underscores safety concerns: indiscriminate activation of pyroptosis can exacerbate systemic inflammation, as exemplified by agents that trigger macrophage pyroptosis and worsen sepsis mortality *in vivo* (Lin et al., 2016). Emerging precision modalities—such as photoactivatable platforms (Zhi et al., 2024), mitochondria/ER-targeted pyroptosis inducers (Lu et al., 2025; Zhi et al., 2024), and gasdermin-selective sdAbs that mitigate off-target inflammatory toxicity (Ma et al., 2023)—collectively suggest that future success will depend on where, when, and in which cells pyroptosis and HMGB1 signaling are modulated (Table 2).

### 4.4 Translational relevance and therapeutic challenges

Despite extensive preclinical validation, clinical translation of pyroptosis–HMGB1 targeting remains nascent: no drug is specifically approved for this axis, though several agents are in early-phase trials. Among HMGB1-targeted approaches, glycyrrhizin has only small-scale human studies, and an anti-HMGB1 antibody (CC001) is in Phase 1/2a for neurodegeneration. Repurposed drugs that modulate pyroptosis—disulfiram (GSDMD inhibitor) in Phase II for Parkinson’s disease and multiple sclerosis, and dimethyl fumarate (FDA-approved for MS)—are being evaluated, while NLRP3 inhibitors (VTX3232, BT-409, HT-6184, dapansutrile, NT-0796) have shown early promise in Parkinson’s disease, myelodysplastic syndromes, and diabetes complications. However, major challenges persist: most inhibitors lack specificity (e.g., glycyrrhizin inhibits 11β-HSD; disulfiram affects aldehyde dehydrogenase), pathway redundancy allows bypass via caspase-3/GSDME or non-pyroptotic HMGB1 release, and systemic inhibition may impair host defense. Additionally, GSDMD inhibitors are ineffective against already-formed pores, and the absence of pharmacodynamic biomarkers (e.g., HMGB1 redox isoforms, GSDMD-NT) hinders patient stratification and target engagement. Future efforts must prioritize isoform-specific assays, combinatorial strategies, precision delivery, and safety studies that preserve beneficial immunity.

## 5 Conclusion and future perspectives

The pyroptosis–HMGB1 axis represents a unifying pathological framework that integrates regulated inflammatory cell death, danger-associated molecular signaling, and immune dysregulation across diverse systemic diseases. Pyroptosis functions not only as a terminal execution program driven by inflammasome activation and gasdermin-mediated membrane permeabilization, but also as a potent amplifier of tissue inflammation through rapid HMGB1 release. In turn, intracellular and extracellular HMGB1 reinforce inflammasome signaling, promote gasdermin execution, and facilitate crosstalk among multiple regulated cell death pathways, thereby establishing a self-sustaining loop of inflammatory injury.

**FIGURE 3:**
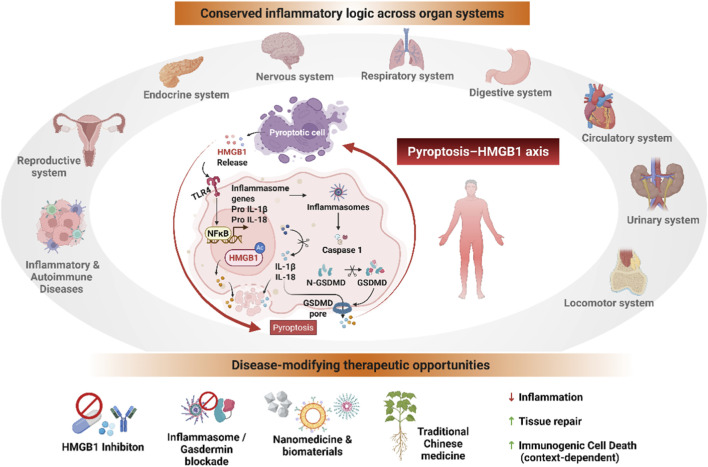
The pyroptosis–HMGB1 axis represents a conserved inflammatory mechanism across multiple organ systems. Inflammasome- and gasdermin-mediated pyroptosis drives the release of HMGB1, which amplifies pattern recognition receptor signaling, cytokine production, and self-sustaining inflammatory loops. This axis functions as a shared pathogenic module in nervous, respiratory, digestive, circulatory, urinary, endocrine, locomotor, reproductive, and autoimmune diseases, despite diverse triggers and tissue contexts. Emerging therapeutic strategies target HMGB1 signaling, the inflammasome–gasdermin execution pathway, and delivery methods, while controlled pyroptosis is highlighted for its context-dependent potential to induce immunogenic cell death in cancer.

Evidence from nervous, respiratory, digestive, circulatory, urinary, locomotor, endocrine, reproductive, and immune-related disorders highlights the recurrent yet context-dependent involvement of this axis. Despite substantial heterogeneity in disease triggers and tissue microenvironments, several shared regulatory principles consistently emerge, including redox imbalance–driven inflammasome priming, cell type–specific gasdermin utilization, and HMGB1-mediated intercellular communication through PRRs such as TLR4 and RAGE. These convergent features support the concept that the pyroptosis–HMGB1 axis reflects a conserved inflammatory logic operating across organ systems, rather than isolated disease-specific mechanisms (Figure 3).

From a therapeutic perspective, a growing repertoire of strategies has been developed to modulate this axis, ranging from direct inhibition of HMGB1 release or activity and suppression of upstream inflammasomes and gasdermins, to targeted delivery approaches enabled by nanomedicine, biomaterials, and multi-target natural compounds. Importantly, these interventions influence not only lytic cell death but also immune activation, vascular integrity, and tissue repair processes, and, in selected contexts, can enhance immunogenic cell death. At the same time, clinical translation faces notable challenges. The dual and context-dependent roles of pyroptosis and HMGB1 require precise temporal, spatial, and cell type–specific control, while extensive crosstalk with apoptosis, ferroptosis, and necroptosis complicates pathway-selective intervention. Pharmacokinetic limitations and delivery specificity further constrain therapeutic application, underscoring the need for biomarker-guided precision strategies and rational combination designs.

Future research should therefore prioritize deeper mechanistic resolution of pyroptosis-HMGB1 interactions, particularly with respect to subcellular localization, post-translational modification of HMGB1, and cell type–specific gasdermin usage. Integration of omics-based biomarkers with functional readouts of pyroptosis and HMGB1 signaling may enable patient stratification and therapeutic monitoring, while continued advances in targeted delivery systems, including nanocarriers, microneedles, and stimuli-responsive biomaterials, offer opportunities to improve specificity and safety. In summary, the pyroptosis–HMGB1 axis constitutes a mechanistically coherent and clinically actionable node linking regulated cell death to immune modulation in systemic diseases, and continued interdisciplinary efforts will be essential to translate this framework into effective and durable therapeutic strategies.
